# Micro-CT reconstruction and structural morphology of the alimentary canal in adult *Tomicus yunnanensis* (Coleoptera: Curculionidae: Scolytinae)

**DOI:** 10.3389/fphys.2026.1922960

**Published:** 2026-09-07

**Authors:** Yuyun Wang, Xinyu Tang, Shunyan Dong, Zongbo Li

**Affiliations:** 1 Key Laboratory of Forest Disaster Warning and Control in Yunnan Province, Southwest Forestry University, Kunming, China; 2 Yunxian Forestry and Grassland Bureau, Lincang, Yunnan, China

**Keywords:** alimentary canal, cryptonephridial system, micro-CT, Scolytinae, ultrastructure

## Abstract

*Tomicus yunnanensis*, the most destructive pest in southwestern China, kills trees by predisposing the host for co-infestation by two sympatric species. Its survival relies largely on diverse capacities of its alimentary canal; thus, micro-computed tomography (micro-CT) and microscopic imaging reveal the morphology and topographical position of the gut, as well as the absence of sex-related differences in total length and regional organisation. The foregut, midgut, and hindgut account for 19.6%, 49.8%, and 30.6% of the total length, respectively. The crop is well developed and bears intimal spines, whereas the proventriculus is highly sclerotized, encircled by robust circular muscles and equipped with cuticular plates and teeth. The midgut is divided into anterior and posterior regions; only the latter possesses gastric caeca. The posterior midgut is structurally similar to the anterior midgut with respect to well-differentiated organelles, but exhibits longer microvilli and more abundant basal folds of the epithelial cells. A cryptonephridial system of six Malpighian tubules, arranged in two groups and attached distally to the anterior rectum, shows distinct proximal-distal structural differences. Epithelial folds forming microvillus-like structures, as well as microfibrils within the endocuticle, vary along the length of the hindgut. These findings are discussed in comparison with the alimentary canal of other Curculionidae (Coleoptera), with a particular focus on the Scolytinae.

## 1 Introduction

The insect alimentary canal, a simple tube from the mouth to the anus, integrates regional structure (e.g., foregut, midgut, and hindgut) and function with gut microbiota to enable digestion, detoxification, nutrient assimilation, immunoregulation, and semiochemical synthesis (Crowson, 1981; Hall et al., 2002; Chapman, 2013; Haider et al., 2025). However, its structural morphology varies significantly with species and diet (Calder, 1989; Chapman, 2013). Coleoptera (>400,000 species), the largest insect order, exhibit diverse feeding habits and are therefore ideal for elucidating gut morphology–function relationships (Crowson, 1981; Beutel et al., 2014). Archostemata, the most primitive suborder, retain a sclerotized proventriculus and a simple hindgut lacking a cryptonephridial system (Crowson, 1981). Algae-feeding Myxophaga possess a simplified proventriculus, a short midgut, and four Malpighian tubules (Yavorskaya et al., 2018). Predatory Adephaga have a large crop, a grinding proventriculus, a short hindgut, and six free Malpighian tubules (Candan et al., 2021). In contrast, Polyphaga, which occupy broad dietary niches, exhibit the greatest morphological diversity, with a variable foregut (crop present or absent), an elongated midgut showing regional differentiation and numerous gastric caeca, and a hindgut that often forms a cryptonephridial system with four or six Malpighian tubules (Calder, 1989; Lopez-Guerrero, 2002; Bu and Chen, 2009; De Sousa and Conte, 2013; Chen et al., 2023; Özyurt Koçakoğlu et al., 2026). Many notorious pests within Polyphaga, including Cerambycidae, Buprestidae, Curculionoidea, and Scolytinae, bore into trees, causing widespread mortality and economic losses. For example, the Asian longhorned beetle (*Anoplophora glabripennis*) has destroyed over 200 million trees in China, caused the felling of about 36,000 trees in Canada, cost the United States over $537 million for eradication effort, and resulted in nearly €20 million in control expenses in Italy’s Lombardy region alone (Wang et al., 2023). Thus, controlling insects that may become pests under certain conditions requires a thorough understanding of their fundmental biology, including alimentary canal, which itself forms the basis of our knowledge of their physiological processes (Crowson, 1981; Díaz et al., 2003; Haider et al., 2025).

*Tomicus yunnanensis* Kirkendall & Faccoli is a univoltine bark beetle belonging to the tribe Hylurgini (Curculionidae: Scolytinae). It is endemic to southwestern China, where it primarily infests Yunnan pine (*Pinus yunnanensis*) (Kirkendall et al., 2008). The species exhibits a typical shoot-boring life cycle (Ye, 1991; Lieutier et al., 2015). Adults emerge from breeding sites in late spring and tunnel into healthy or weakened shoots for maturation feeding, causing shoot wilting, breakage, and crown dieback. After maturation feeding over half a year, numerous individuals, including two sympatric species, *T. minor* and *T. brevipilosus*, jointly predispose on weakened host trees guided by chemical cues, primarily terpenoid compounds (Liu et al., 2010, 2025) and aggregation pheromones (Wu et al., 2019), following a top-down attack pattern that shifts from shoots to stems (Liu et al., 2019). After mating, females oviposit under the bark, and larvae feed on phloem, creating offspring galleries nearly perpendicular to the maternal gallery, which disrupts nutrient and water transport and ultimately leads to tree mortality. Newly emerged adults then fly to adjacent pine crowns to initiate the next generation. Reproduction is strongly influenced by host condition, temperature, and intraspecific competition (Ye, 1991; 1994; Lu et al., 2012), with population outbreaks typically associated with drought-stressed hosts (Yu et al., 2022). Geographically, *T. yunnanensis* is distributed across the Yunnan-Guizhou Plateau, including Yunnan, Sichuan, Guizhou, and southern Gansu provinces, and its range is expanding due to climate warming and human activity. Since the 1980s, continuous outbreaks have caused widespread Yunnan pine mortality, with 1.5 million hectares of trees killed in Yunnan alone (Ye, 1991; Yu et al., 2022). In severely affected areas, such as the Kunming region and the upper Yangtze River reaches, tree mortality rates can exceed 50–70% in heavily infested stand, resulting in substantial economic losses, reduced watershed water-holding capacity, biodiversity loss, and increased fire risk.

Currently, these ecological and economic impacts have prompted intensive research into integrated pest management for *T. yunnanensis*, including semiochemical-based monitoring, biological control using local parasitoids (e.g., *Coeloides* spp.), and mixed-species plantations and timely removal of infested trees. Nevertheless, the beetle’s population and range continue to expand (Yu et al., 2022). Although information on *T. yunnanensis* is abundant, essential knowledge, particularly the morphology and structure of its alimentary canal, which is critical for understanding physiology and for enabling potential pest control applications, remains limited. Detailed studies on the alimentary canal of scolytids exist for only a dozen bark beetle species (Baker and Estrin, 1974; Calder, 1989; Hallberg, 1993; Díaz et al., 1998, 2000; Hall et al., 2002; Nardi et al., 2002; Díaz et al., 2003; Silva-Olivares et al., 2003; Bu and Chen, 2009) and several ambrosia beetles (Schneider and Rudinsky, 1969). These studies reveal general anatomical and histological similarities across scolytids, yet species-specific morphological adaptations related to bark feeding and symbiotic interactions also exist. For example, *Dendroctonus ponderosae* exhibits regional midgut differentiation with numerous gastric caeca enhancing nutrient absorption (Díaz et al., 2003); *D. armandi* possesses a highly sclerotized proventriculus with eight chitinous plates for grinding wood particles (Bu and Chen, 2009); *Ips typographus* shows a well-developed cryptonephridial system in which Malpighian tubules attach to the hindgut, improving water conservation (Hallberg, 1993). Given that *T. yunnanensis* feeds on both pine shoots and phloem, a diet distinct from the phloem-only and fungus-only diets of the aforementioned scolytids (Kirkendall et al., 2015), further investigation into its digestive structure is essential.

The present study aims to: (1) visualise the general morphology and subdivisions of the alimentary canal using micro-computed tomography (micro-CT); (2) characterise the morphological structures of each gut region using light, scanning and transmission electron microscopy; and (3) identify morpho-functional adaptations, with particular emphasis on the anterior midgut, by comparing *T. yunnanensis* with other known scolytid beetles. This study will serve as a basis for future research and advance current knowledge of the alimentary canal across Coleoptera.

## 2 Material and methods

### 2.1 Insect collection

Adult *Tomicus* beetles were collected from infested *P. yunnanensis* shoots in October at Jiulongshan Forest Farm, Qujing, Yunnan, China, and promptly transported to our laboratory at Southwest Forestry University in Kunming. Species identification among the three sympatric *Tomicus* species, *T. yunnanensis*, *T. minor*, and *T. brevipilosus*, was performed primarily by the arrangement of punctures on the second elytral interstria (biseriate or irregularly uniseriate) and the puncture spacing in adjacent striae (Kirkendall et al., 2008). Following species identification, the sex of *T. yunnanensis* adults was determined by the morphology of the eighth abdominal tergite or by stridulation (males chirp, whereas females are silent). All sexed adults were temporarily stored at 4°C, usually for no more than three days.

### 2.2 Micro-CT imaging

Following the protocol of Alba-Alejandre et al. (2019), beetles stored at 4 °C were killed by immersion in 30% ethanol for 25 min. Surface debris was removed by ultrasonic treatment in chilled phosphate-buffered saline (0.01 M, pH 7.4) at 40 kHz for 5 min. Specimens were then fixed in Bouin’s solution for 48 h at room temperature, after which the fixative was replaced with 1% (w/v) iodine in absolute ethanol for staining in the dark for 48–72 h. Samples were rinsed 3–5 times with ultrapure water and completely air-dried under ventilated, dust-free conditions. Each individual dried beetle was mounted inside a pipette tip and subsequently scanned with an Xradia 520 Versa X-ray microscope (ZEISS, Jena, Germany). Scanning parameters were: voltage 40 kV, current 75 µA; isotropic voxel size 2.57 µm (female) and 2.97 µm (male); 360° rotation scanning in HART mode with step sizes of 0.3° (male) and 0.4° (female). This generated 4,203 images (1,401 images/segment × 3 scans) per specimen (2 s/image; total scan time 4 h 24 min). Primary 3D reconstruction was performed using the micro-CT’s built-in Scout-and-Scan Control System, with duplicate sections removed during the stitching process, ultimately generating TIFF image stacks (2,199 slices for the female, 2,073 for the male). The datasets were imported into Amira 3D (Version 2024.1, Thermo Fisher Scientific, Waltham, MA, USA) for processing and visualization. After volume rendering, individual structures were segmented using a watershed algorithm, each assigned a unique label. To preserve original texture while applying target colors, the operation A × B was used, where A represents the segmented tissue and B the color vector from the “labels.am” color table. A total of three individuals per sex were examined. Measurements of length and surface area of the alimentary canal and its subdivisions were taken manually from the mouth to the anus using Amira. Sex-related differences were analyzed by independent samples t-test in R software (v4.4.1; https://cran.r-project.org/).

### 2.3 Scanning electron microscopy

Ten dissected alimentary canals and ten proventriculi (5 per sex) were fixed in 2.5% glutaraldehyde and 2% paraformaldehyde in 0.1 M PBS at 4 °C for 12 h. After dehydration through a graded ethanol series (50%, 70%, 80%, 90%, 95%, and absolute ethanol) for 10 min per step and two 15-min washes in xylene, the samples (intact and specific injured regions) were critical-point dried (Quorum K850, Ashford, Kent, UK), mounted on stubs, and sputter-coated with gold for 300 s using a Cressington 108 auto coater (Chalk Hill, Watford, UK). Observations and imaging were performed using a ZEISS Sigma 300 SEM (ZEISS, Jena, Germany) at an accelerating voltage of 7 kV.

### 2.4 Transmission electron microscopy

Ten guts (5 per sex) were fixed in 2.5% glutaraldehyde and 2% paraformaldehyde in 0.1 M PBS. To enhance fixation and prevent tissue degradation, samples were vacuum-infiltrated for 20min using a syringe and then fixed overnight at 4 °C. Specimens were post-fixed in 1% osmium tetroxide at 4 °C for 1.5 h, dehydrated through the above-mentioned ethanol series for 5min per step, and infiltrated with 3:1, 1:1, and 1:3 ethanol/Epon 812 mixtures for 2, 4, and 12 h, respectively, followed by pure Epon 812 resin. After infiltration, specimens were positioned under a stereomicroscope and polymerized at 30 °C and then 60 °C for 24 h each. Excess resin was trimmed away. For precise localization of gut subdivisions, semi-thin sections (approx. 800 nm) were cut using a Leica EM UC7 ultramicrotome (Leica, Wetzlar, Germany) and stained with toluidine blue for 15 s, and photographed under a BX-51 light microscope (Olympus, Tokyo, Japan). Ultrathin sections (approx. 70 nm) were then cut, stained with 2% uranyl acetate (8 min) and 0.4 % lead citrate (10 min), and examined using a Hitachi JEM-1400 plus TEM.

## 3 Results

### 3.1 General morphology of the alimentary canal

**Figure 1:**
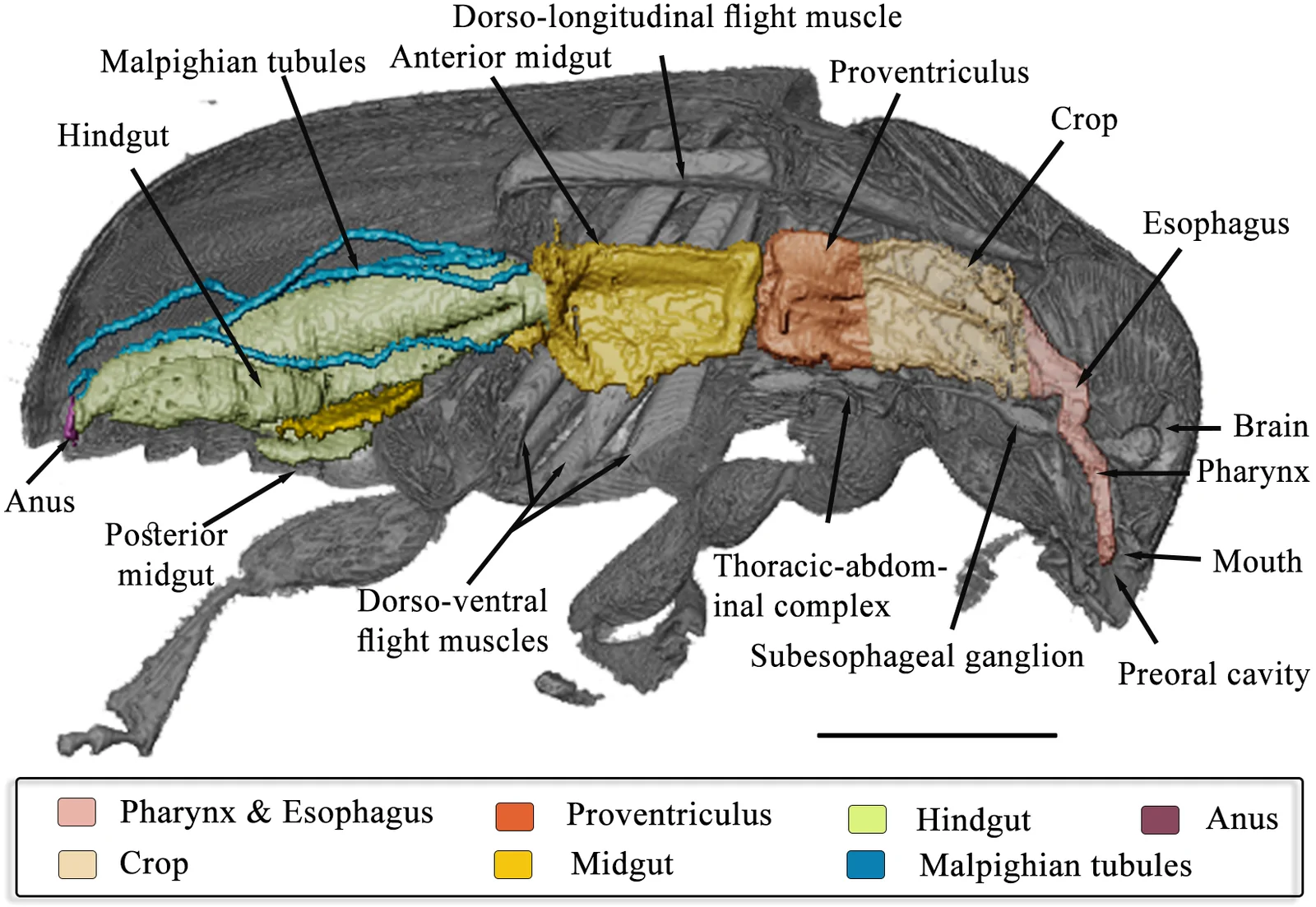
Morphology and regional subdivisions of the alimentary canal of *Tomicus yunnanensis* in right-lateral aspect. Scale bar = 1000 μm.

**Figure 2:**
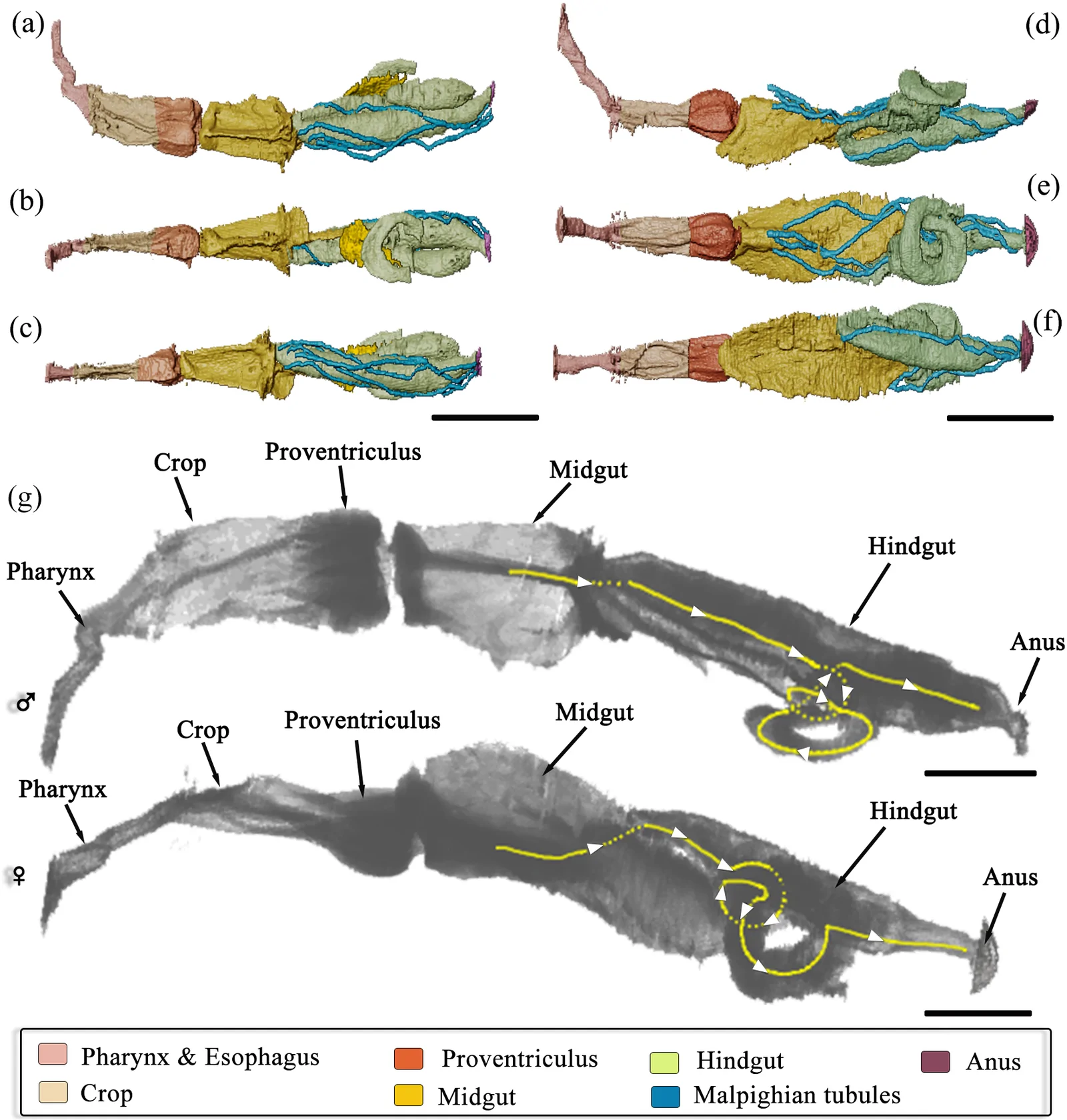
Alimentary canal of a male **(a–c)** and a female **(d–f)** *Tomicus yunnanensis* in right-lateral **(a, d)**, ventral **(b, e)**, and dorsal **(c, f)** views. **(g)** Alimentary canals with the trajectories and looping patterns of the midgut and hindgut are delineated by yellow lines and black arrowhead. Scale bar: **(a–f)** = 1000 μm; (g) = 500 μm.

**Figure 3:**
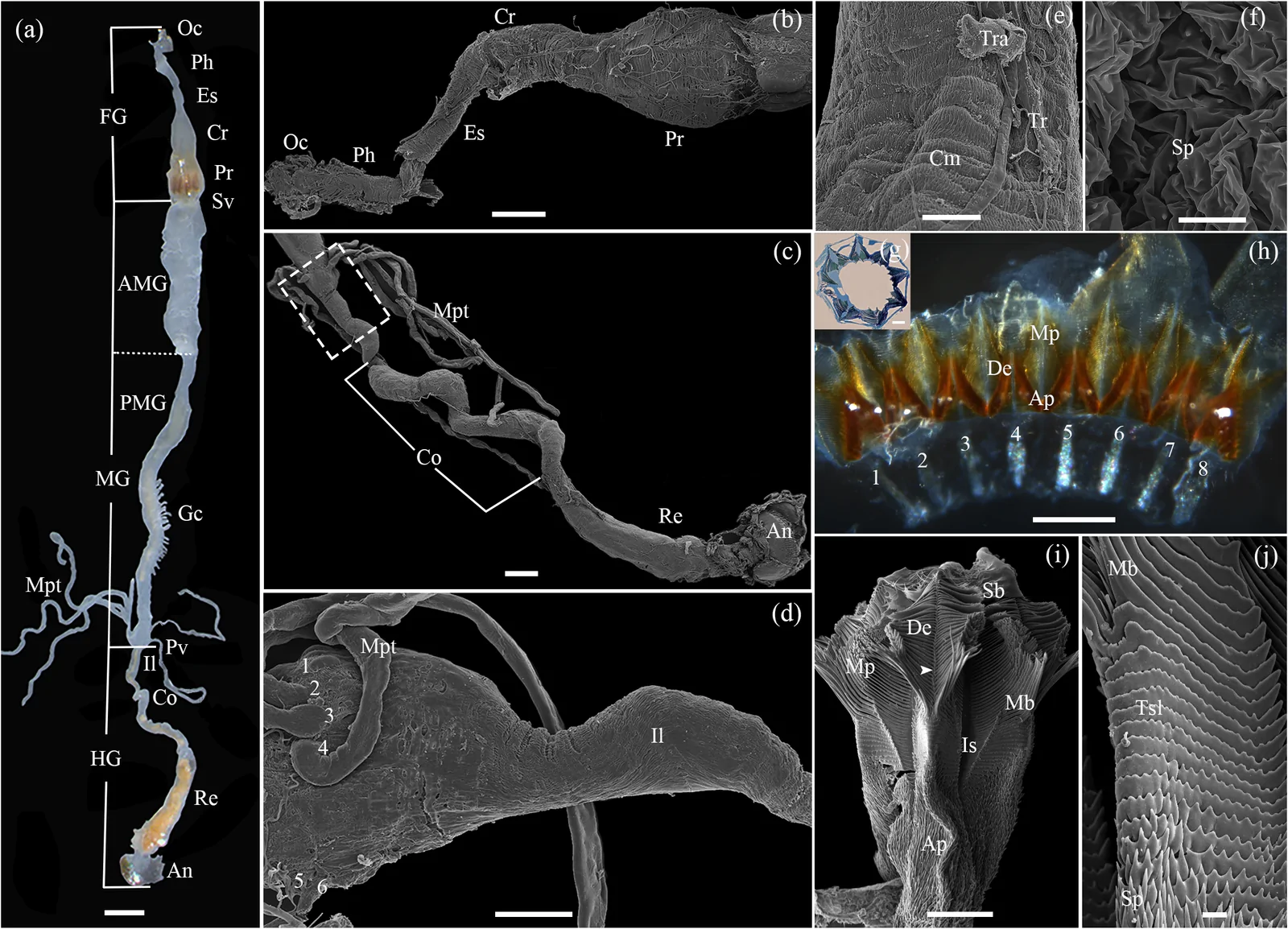
Morphology of the alimentary canal in *Tomicus yunnanensis*. **(a)** Overall morphology; **(b)** Anterior foregut; **(c)** Entire hindgut, showing Malpighian tubule insertion (dashed box, magnified in **(d)** and subdivision morphology; **(d)** Magnified insertion site, showing four upper and two lower tubules; **(e)** Magnified crop surface, showing tracheae and tracheoles over circular muscles; **(f)** Folds and spines of the crop intima; **(g)** Octagonal outline of the proventriculus under transverse section; **(h)** Numbered chitinous plates of the proventriculus visualized by light microscopy; **(i)** Internal view of the proventricular chitinous plates; **(j)** Magnified junction between the anterior plate and the masticatory plate. An, anus; AMG, anterior midgut; Ap, anterior plate; Cm, circular muscle; Co, colon; Cr, crop; De, declivity; Es, esophagus; FG, foregut; Gc, gastric caeca; HG, hindgut; Il, ileum; Is, intermediate suture; Mb, masticatory brushes; MG, midgut; Mp, masticatory plate; Mpt, Malpighian tubules; Oc, oral cavity; Ph, pharynx; PMG, posterior midgut; Pr, proventriculus; Pv, pyloric valve; Re, rectum; Sb, stopping bristles; Sp, spine; Sv, stomodeal valve; Tr, tracheoles; Tra, tracheae; Tsl, transverse spine lines. Scale bars: **(a, b, c, g)** = 200 µm; **(d, f, h, i)** = 10 µm; **(e)** = 50 µm.

The alimentary canal of *T. yunnanensis* is a convoluted tube positioned centrally within the hemocoel, extending from the mouth to the anus (**Figure 1**). No sexual differences are observed in overall morphology, regional lengths, and looping patterns (**Figures 2a–g**; all P > 0.05; see **Table 1** for values). Unless otherwise indicated, all subsequent gut descriptions (e.g., measurements, morphology, and structural features) therefore refer to characteristics common to both sexes. Three gut regions are separated by two valve-like folds: the stomodeal valve (foregut–midgut) and the pyloric valve (midgut–hindgut) (**Figure 3a**). Micro-CT imaging estimates gut length as approximately equal to body length, whereas dissection reveals a length two to five times longer (**Table 1**), thereby reflecting the gut’s true folded state within the body cavity. Based on mean values derived from dissections of both sexes, the foregut, midgut, and hindgut account for 19.6%, 49.8%, and 30.6% of the total alimentary canal length, respectively.

**Table 1:** Morphometric measurements of the alimentary canal of *Tomicus yunnanensis* obtained by micro-CT (n = 3 per sex) and microdissection (n = 10 per sex), with all data expressed as mean ± standard deviation.

Alimentary canal	Micro-CT		Microdissection	
	Female (mm)	Male (mm)	Female (mm)	Male (mm)
Total	5.63±0.51	5.12±0.53	15.72±2.48	15.11±2.16
Foregut	2.36±0.08	2.18±0.14	2.85±0.22	3.06±0.25
midgut	1.34±0.18	1.22±0.14	7.81±0.35	7.62±0.63
hindgut	1.93±0.19	2.10±0.12	5.05±0.21	4.97±0.09
Malpighian tubules	2.18±0.08	2.84±0.11	3.92±0.11	3.96±0.13

### 3.2 Foregut

The foregut extends from the mouth to the prothorax and comprises a narrow anterior tube (pharynx and esophagus) and an enlarged posterior region (crop and proventriculus) (**Figure 1**; **2g**; **3a, b**). The pharynx is a straight tube lined with chitinized ridges, where the pharyngeal dilator muscles insert and open into the buccal cavity. This cavity traverses the head, passes below the brain, and connects to the crop via a short esophagus. The crop is spindle-shaped, broadest medially and tapering toward the proventriculus. Its circular muscles bear numerous tracheae and tracheoles, and its intima is lined with abundant spines (**Figures 3e, f**). The proventriculus is an octagonal organ surrounded by outer circular muscles that envelop eight heavily sclerotized plates (**Figures 3g-j**). These grinding plates, each consisting of anterior and masticatory portions, form a star-like outline in transverse section. Transverse lines of spines on the anterior plates triturate food particles, while stopping bristles project into the lumen to filter particles and prevent retrograde flow. The epithelium gradually increases in thickness from the pharynx (15.3 ± 5.6 µm) to the proventriculus (91.9 ± 18.4 µm) and dynamically modulates luminal volume by folding inward when empty and flattening when filled, as clearly shown by micro-CT imaging (**Supplementary Figure 1**).

**Figure 4:**
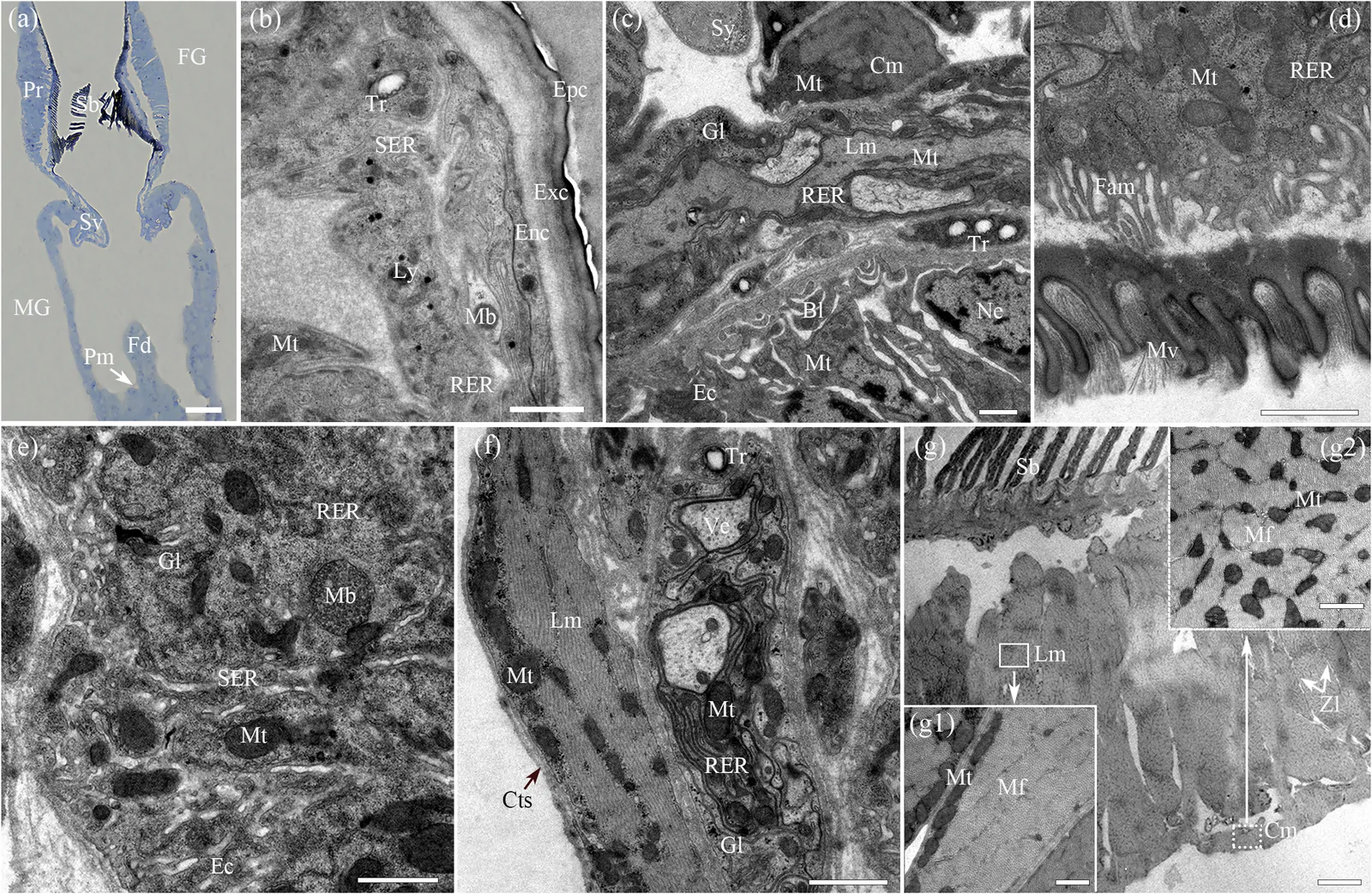
Internal structure of foregut subdivisions in *Tomicus yunnanensis*. **(a)** Location and morphology of the proventriculus, also showing the stomodeal valve and anterior midgut; **(b)** Trilayered cuticular intima of the esophagus: endocuticle, exocuticle, and epicuticle; **(c)** Circular muscle layer and epithelial cells of the proventriculus; **(d)** Folds of the apical cytoplasmic membrane in the stomodeal valve, showing numerous microvilli; **(e)** Epithelial cell of the stomodeal valve; **(f)** Circular muscle and adjacent epithelial cell in the stomodeal valve; **(g)** Proventricular cuticle, highlighting two types of cuticular muscle (box). Bl, basal lamina; Cm, circular muscle; Cts, connective tissue sheath; Ec, epithelial cell; Enc, endocuticle; Epc, epicuticle; Exc, exocuticle; Fam, folds of the apical cytoplasmic membrane; Fd, food; FG, foregut; Gl, glycogen; Lm, longitudinal muscle; Ly, lysosome; Mb, multivesicular body; Mf, myofilament; MG, midgut; Mv, microvilli; Ne, nucleus; Pm, peritrophic membrane; Pr, proventriculus; RER, rough endoplasmic reticulum; Sb, stopping bristles; SER, smooth endoplasmic reticulum; Sv, stomodeal valve; Sy, synapse; Tr, tracheoles; Ve, vesicles; Zl, Z-line. Scalebar:(a) =100 µm; (b-f, g1-g2) = 1 µm; **(g)**=10 µm.

From the lumen outward, the foregut wall consists of three layers: a cuticular intima, a single layer of epithelial cells, and two muscle layers, including an inner longitudinal layer and an outer circular layer (**Figures 4a–g**). The cuticular intima is a thick structure comprising three distinct layers from the innermost to the outermost: the epicuticle, exocuticle, and endocuticle (**Figure 4b**). These three layers are equal in thickness, measuring 193.8 ± 46.3 nm, 218.8 ± 20.8 nm, and 234.3 ± 29.4 nm, respectively; however, the epicuticle is more electron-dense than the endocuticle and exocuticle. The epithelial cells lie on a continuous basal lamina situated beneath the longitudinal muscle layer. The basal plasma membrane occasionally shows deep invaginations (**Figure 4c**). Glycogen granules are abundant in the basal region, with rough endoplasmic reticulum (RER) beneath them and closely associated with the elongated nucleus. Mitochondria are interspersed among the RER cisternae. The middle region contains many mitochondria and bundles of smooth endoplasmic reticulum (SER), with lysosomes and multivesicular bodies occasionally observed (**Figures 4b, e**). In longitudinal sections, the SER displays a tubular morphology. Mitochondria also cluster apically, surrounding the cuticular intima. The apical cytoplasmic membrane exhibits cup-like foldings, and the luminal aspect of these structures is covered with short, sparse microvilli (**Figure 4d**). The longitudinal muscles lie adjacent to the epithelium, whereas circular muscles occupy the outermost layer. In longitudinal sections, the longitudinal muscle layer appears continuous, whereas the circular muscle layer is only intermittently visible (**Figures 4c, f, g**). Epithelial and longitudinal muscle cells contain oval nuclei, whereas circular muscle cells have elongated nuclei. A connective tissue sheath surrounds the entire muscularis (**Figure 4f**). The muscles are of the visceral striated type, with myofibril-rich cytoplasm and regularly spaced Z-lines. Mitochondria are mainly located at the cell periphery, near the sarcolemma, though their distribution pattern varies (**Figure 4g1–g2**). Axon terminals form synapses on the longitudinal muscle surface, characterised by electron-dense granules and mitochondria (**Figure 4c**).

However, each foregut subdivision shows certain changes in muscle thickness, epithelial folding, and cuticular spines (**Figures 4a, b, d, f, g**). The pharynx has a thin cuticle and prominent longitudinal folds. The esophagus exhibits both longitudinal and transverse folds, along with small spines. In the crop, epithelial folds penetrate the muscle layer, forming non-sclerotized grooves lined with numerous spines (**Figure 3f**). The proventriculus shows the most pronounced variation: its folds extend into the circular muscle layer, creating sclerotized grooves with short, thick spines (**Figures 3i, j**; **4a, g**). Among all foregut regions, the proventriculus possesses the thickest muscle layer (approx. 78 µm), compared with the thinnest (approx. 0.4 µm) observed in the stomodeal valve (**Figures 4f, g**). Its epithelial cells contain large, oval nuclei and cytoplasm rich in RER and glycogen granules, while the intima appears as a highly electron-dense, wavy to lamellar structure.

### 3.3 Midgut

**Figure 5:**
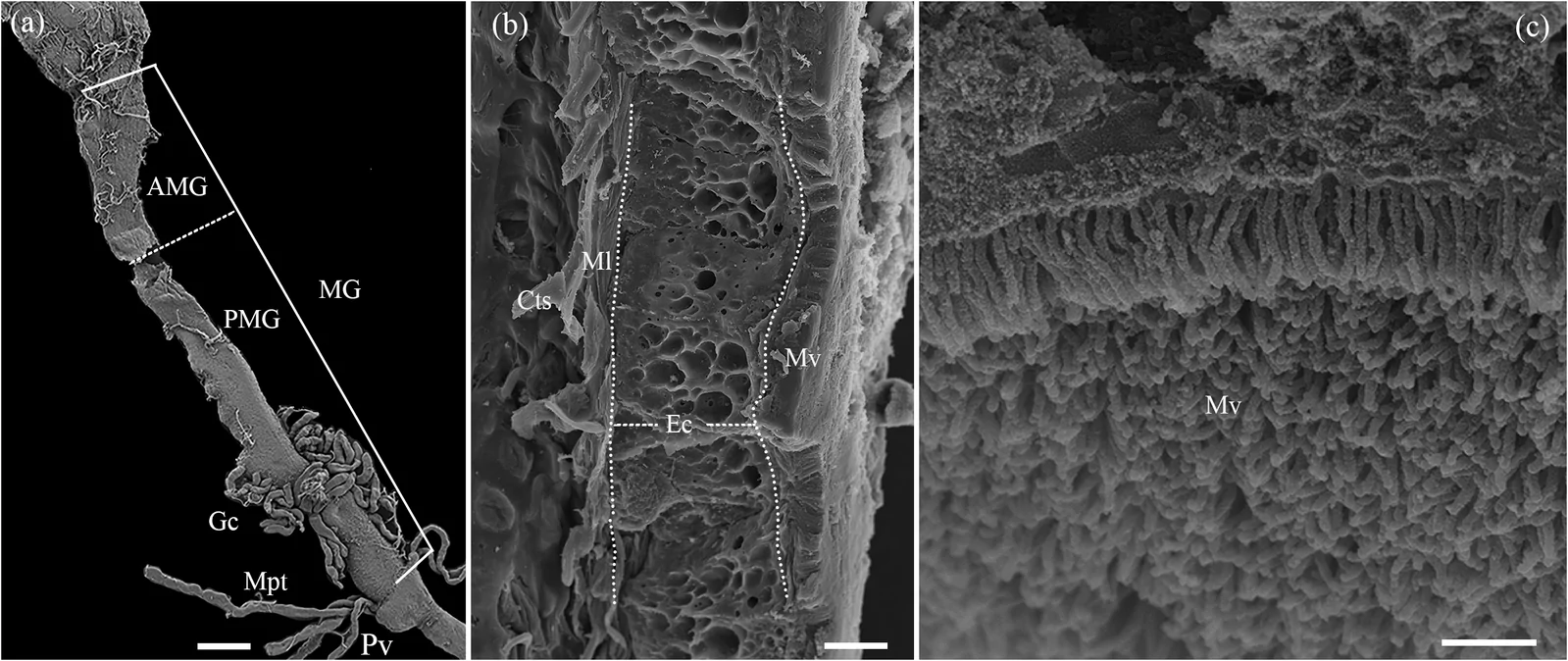
Morphology of the midgut in *Tomicus yunnanensis*. **(a)** Overall morphology, showing a fragile connection between the anterior and posterior midgut; **(b)** Fracture surface of the midgut, revealing two layers (dotted line): a thin muscle layer and an epithelial layer with numerous microvilli; **(c)** Fine morphology of microvilli. AMG, anterior midgut; Cts, connective tissue sheath; Ec, epithelial cell; Gc, gastric caeca; MG, midgut; Ml, muscle layer; MV, microvilli; PMG, posterior midgut; Pv, pyloric valve. Scale bar: **(a)** = 200 µm; **(b)** = 5 µm; **(c)** = 500 nm.

The midgut exhibits a markedly convoluted morphology that spans the mesothorax, metathorax, and part of the abdomen (**Figure 1**; **3a**). It is divided into two distinct regions: a short, ovoid, wide anterior portion (1400.31 ± 208.15 µm) and a long, narrow posterior part (2489.45 ± 509.49 µm). The anterior midgut appears more delicate under light microscopy and is more fragile than the posterior midgut during the drying process (**Figure 3a**; **5a**).

**Figure 6:**
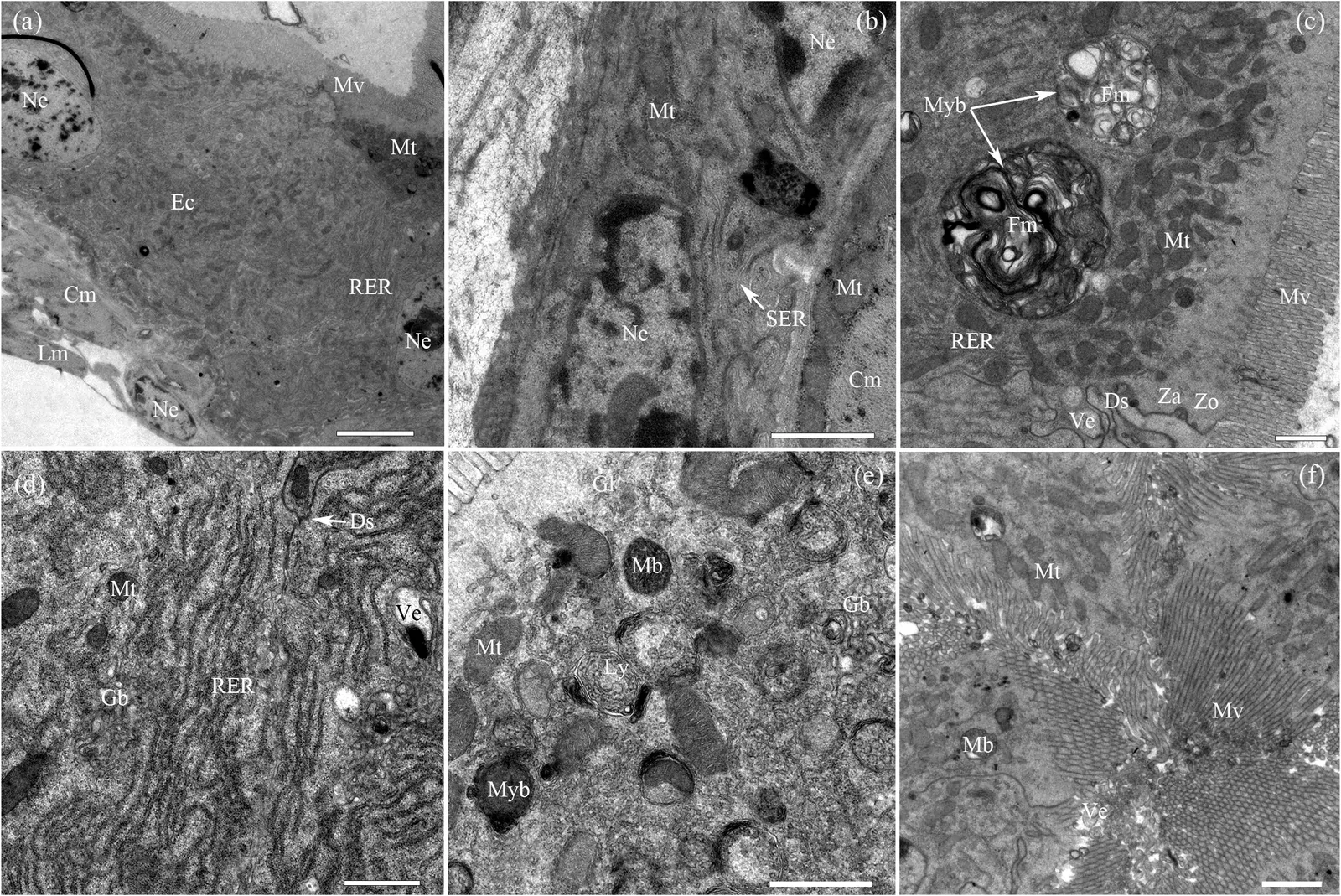
Internal structure of the anterior midgut in *Tomicus yunnanensis*. **(a)** Cuticular layer components, showing a muscle layer and an epithelial cell with numerous microvilli; **(b-f)** Organelle distribution in anterior midgut cells: circular muscle and adjacent cellular nidi **(b)**, mid-apical epithelial cells **(c)**, rough endoplasmic reticulum **(d)**, apical cell **(e)**, and apical extrusions into the midgut lumen **(f)**. Cm, circular muscle; Ds, desmosome; Ec, epithelial cells; Fm, flocculent material; Gl, glycogen; Gb, Golgi body; Ly, lysosome; Mb, multivesicular body; Mt, mitochondria; Mv, microvilli; Myb, myeloid bodies; Ne, nucleus; RER, rough endoplasmic reticulum; SER, smooth endoplasmic reticulum; Ve, vesicles; Za, zonula adherens; Zo, zonule occlusive. Scale bar: **(a)** = 5 µm; **(b-e)** = 1 µm.

Structurally, the midgut resembles the foregut, both consisting of three layers: a peritrophic membrane, a monolayer of epithelial cells, and two muscle layers (**Figure 6a**). Unlike the foregut intima, however, the midgut peritrophic membrane is a fibrous structure that encloses the food bolus, thereby protecting the epithelium from food particles and microorganisms (**Figure 4a**; **Supplementary Figure 2**). The midgut epithelium is of reduced thickness (17.1 ± 0.5 µm) and composed of large, vacuolated cells bearing abundant microvilli (**Figures 5b, c**). Additionally, the longitudinal muscle layer lies external to the circular muscle layer, and both are thin (**Figures 6a, b**). The vacuolated cells are distinctly classified into two types: columnar cells and regenerative cells (nidi). In the anterior midgut epithelium, three distinct types of junctional complexes occur on lateral cell surfaces of adjacent cells (**Figure 6c**). At the apical region, a smooth septate junction (zonula occludens) is present between neighboring cells, followed basally by zonula adherens; desmosomes are found at various sites along the lateral membranes (**Figure 6d**). The apical surfaces bear microvilli with vesicles at their tips. The basal microvillar region exhibits a brush-like border formed by irregular, electron-dense glycogen deposits, while the area adjacent to this border contains numerous mitochondria together with flocculent or laminated myeloid bodies (**Figures 6c, e, f**). Lysosomes are single-membrane bound: some are round and uniformly dense, others irregular with variable electron density. Ribosomes are abundant throughout the cytoplasm, either free or as components of the RER. The RER cisternae are usually arranged in parallel (**Figure 6d**) occasionally appear interwoven with spiral SER (**Supplementary Figure 3**). The basal membrane invaginates to form the basal labyrinth, which varies in length and thickness. Columnar cells have a central, ovoid nucleus with dispersed euchromatin. In contrast, nidi cells possess an irregular contour and a round to ovoid nucleus with heterogeneous clumps; their cytoplasm contains scarce SER, abundant free ribosomes, and small mitochondria. Nidi cells lack junctional structures, secretory vesicles, and the basal labyrinth (**Figure 6b**).

**Figure 7:**
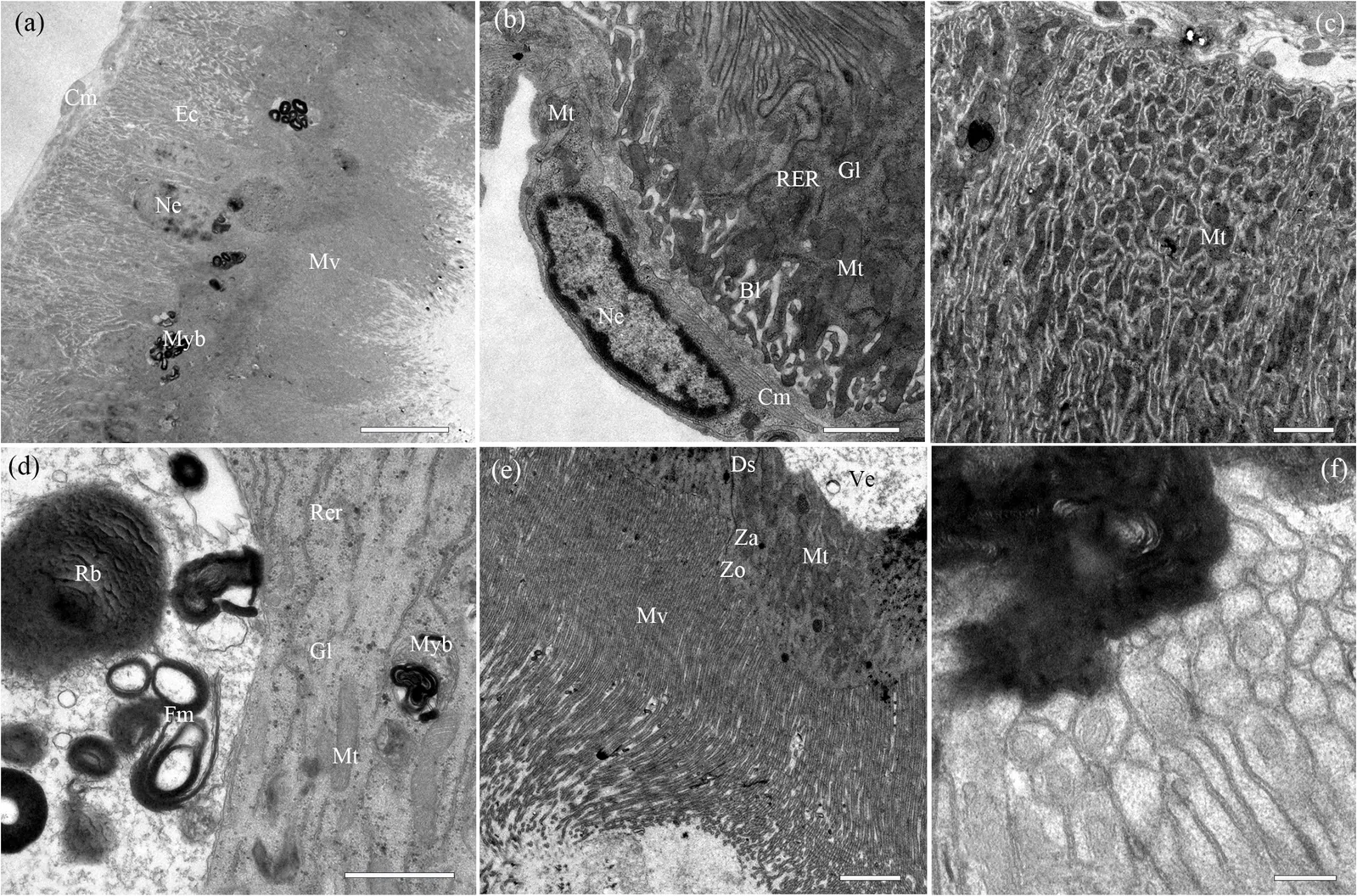
Internal structure of the posterior midgut in *Tomicus yunnanensis*. **(a)** Cuticular layer components, showing a muscle layer and an epithelial cell with numerous elongated microvilli; **(b)** Organelle distribution in posterior midgut cells: circular muscle and basal lamina **(b)**, basal labyrinth **(c)**, middle epithelial cells containing myeloid bodies and residual bodies **(d)**, apical surface **(e)**, and secretory vesicles released into the midgut lumen **(f)**. Bl, basal lamina; Cm, circular muscle; Ds, desmosome; Ec, epithelial cells; Fm, flocculent material; Gl, glycogen; Mt, mitochondria; Mv, microvilli; Myb, myeloid bodies; Ne, nucleus; Rb, residual body; RER, rough endoplasmic reticulum; Ve, vesicles; Za, zonula adherens; Zo, zonule occlusive. Scale bar: **(a)** = 10 µm; **(b, d)** = 1 µm; **(c, e)** = 2 µm; **(f)** = 200 nm.

The epithelial cells of the posterior midgut, including their junctional complexes, glycogen granules, nuclei, lysosomes, secretory vesicles, ribosomes, and basal labyrinth, closely resemble those of the anterior midgut (**Figures 7a–f**). Similarly, near the mid-region of the cells, large, oval, flocculent or laminated myeloid bodies, electron-lucent vesicles, fine granules, and large nuclei with heterochromatin clumps are also present (**Figures 7d, f**). However, the microvilli of the columnar cells are markedly longer (17.9 ± 3.3 µm) than those in the anterior midgut (1.8 ± 0.4 µm). In the same region, the RER and the folds of the basal labyrinth are more abundant, and these folds create an extracellular space between the basal plasma membrane and the basal lamina (**Figure 7c**).

**Figure 8:**
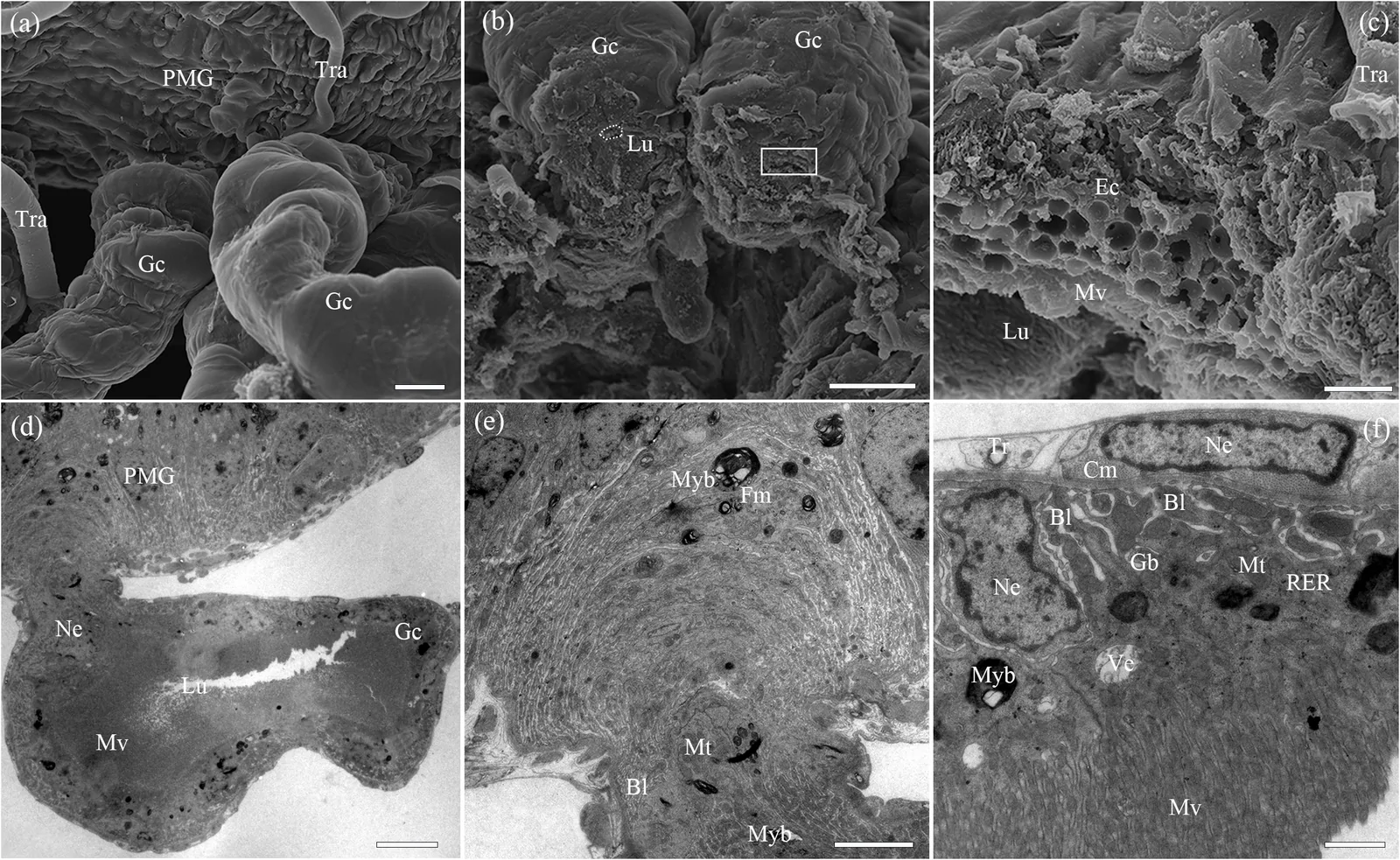
Morphology and internal structure of gastric caeca in *Tomicus yunnanensis*. **(a)** Junction between gastric caeca and posterior midgut; **(b)** Fractured surface of a gastric caecum, showing a central lumen (dotted circle, solid box magnified in c); **(c)** Cuticle of gastric caecum, showing an epithelial layer with microvilli projecting into the lumen; **(d)** Transverse section of the gastric caeca–posterior midgut junction; **(e)** Magnified view of the junction; **(f)** Cuticle of gastric caecum, showing a thin circular muscle layer and an epithelial cell. Bl, basal lamina; Cm, circular muscle; Ec, epithelial cell; Ne, nucleus; Fm, flocculent material; Gb, Golgi body; Gc, gastric caeca; Lu, lumen; Mt, mitochondria; Mv, microvilli; Myb, myeloid bodies; Ne, nucleus; PMG, posterior midgut; RER, rough endoplasmic reticulum; Tr, tracheoles; Tra, trachea; Ve, vesicles. Scale bar, **(a, b, d)** = 10 µm; **(c)** = 2 µm; **(e)** = 5 µm; **(f)** = 1 µm.

Gastric caeca are located in the middle region of the posterior midgut (**Figures 3a**, **5a**). These structures are short, curly, tube-like projections that protrude outward from the midgut surface and are arranged in two rows (**Figures 8a-c**). Each caecum measures 200.82 ± 32.67 µm in length, and the total number of caeca is 42. Internally, each gastric caecum consists of a basal region and an apical region (**Figure 8d**). The basal region is composed of columnar cells, whose cytoplasm contains RER, mitochondria, myeloid bodies, and microvilli extending from the apical membrane (**Figure 8e**). The apical region is characterised by cells with basally located nuclei, along with mitochondria, RER, lipid droplets, and sac-like vesicles (**Figure 8c**).

### 3.4 Hindgut

**Figure 9:**
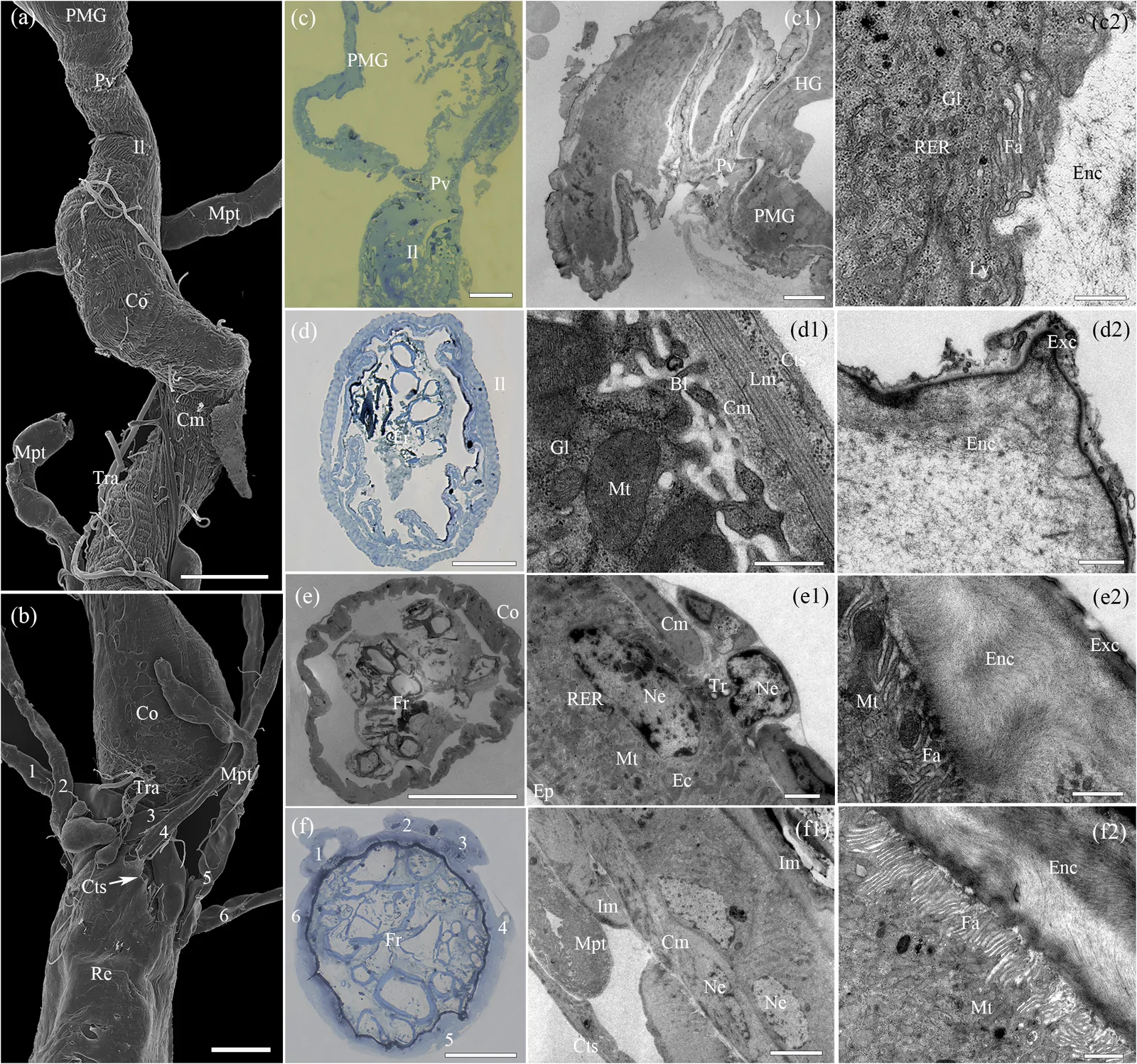
Morphology and internal structure of hindgut subdivisions in *Tomicus yunnanensis*. **(a)** Morphology of the anterior hindgut; **(b)** Junction between colon and rectum, showing six Malpighian tubules (numbered) inserting into the anterior rectum; **(c)** Pyloric valve position, with overall (c1) and inner (c2) views; **(d)** Transverse section of ileum, with outer (d1) and inner (d2) views; **(e)** Transverse section of colon, with outer (e1) and inner (e2) views; **(f)** Transverse section of anterior rectum, with outer (f1) and inner (f2) views. Bl, basal lamina; Cm, circular muscle; Co, colon; Cts, connective tissue sheath; Ec, epithelial cell; Enc, endocuticle; Exc, exocuticle; Fa, folds of the apical cytoplasmic membrane; Fr, food residuals; Gl, glycogen; HG, hindgut; Il, ileum; Im, internal membrane; Lm, longitudinal muscle; Ly, lysosome; Mpt, Malpighian tubule; Mt, mitochondria; Ne, Necleus; PMG, posterior midgut; Pv, pyloric valve; Re, rectum; RER, rough endoplasmic reticulum; Tr, tracheole; Tra, trachea. Scale bars, (a–f) = 100 µm; (c1) = 10 µm; (f1) = 5 µm; (e1) = 2 µm; (f2) = 1 µm; (c2, d1, d2, e2) = 500 nm.

The hindgut begins at the insertion of the Malpighian tubules and is a simple, straight, narrow tube subdivided into the ileum, colon, and rectum (**Figures 3a, c, d**; **9a, b**). Yellow food residues are clearly visible in the lumen of each subdivision. The ileum gradually transitions into the colon without a clear posterior boundary; the colon is a short, straight tube leading to the rectum. The rectum is an enlarged, sclerotized section often containing accumulated yellow residues. The Malpighian tubules attach to the proximal rectum (**Figure 9b**), and the rectum opens to the exterior through the anus at the posterior end of the abdomen. The pyloric valve, composed of short epithelial folds protruding into the ileal lumen, marks midgut–hindgut transition (**Figure 9c, c1**). These folds narrow the ileal lumen. In the transitional region, the posterior midgut epithelium has narrower cells whose apical microvilli shorten and disappear near the valve. In contrast, the hindgut epithelium cells are columnar but flattened, with folds of apical plasma membrane bearing very short microvilli; the underlying cytoplasm contains numerous mitochondria, ribosomes, RER, and a few lysosomes (**Figure 9c2**).

Furthermore, the three hindgut subdivisions exhibit a structure similar to that of the foregut, consisting of an intima, an epithelial cell, and two muscle layers (**Figures 9d-f**). Their epithelium is slightly thicker than that of the midgut, measuring 24.2 ± 7.1 µm, 25.9 ± 9.1 µm, and 26.2 ± 5.4 µm, respectively. The intima is thin, which facilitates the permeation of water and inorganic salts. The exocuticle is thin and electron-dense, whereas the endocuticle is thicker, less electron dense, and contains one or two layers of microfibrils that become more prominent from the ileum to the rectum (**Figure 9d2, e2, f2**). At the apical region of the columnar epithelial cells, folds of the cytoplasmic membrane form microvillus-like structures that similarly become more distinct from the ileum to the rectum. Mitochondria and RER are scattered throughout the cytoplasm but are less abundant than in the midgut epithelium, with no specific distribution (**Figure 9c2, d1, e2, f1**). The ovoid nucleus is basally located with heterochromatin clumps. Lysosomes are few, round or irregular, with variable electron density. The muscle layer arrangement mirrors that of the midgut: circular muscle internally, longitudinal muscle externally (**Figure 9d1**). Muscle thickness varies, consisting of several rows of circularly arranged myocytes or a single thick row (**Figure 9d1, e1**). A thin connective tissue layer with a thickness of approximately 0.50 ± 0.14 µm surrounds the external muscle layer (**Figure 9b, d1, f1**).

### 3.5 Malpighian tubules

**Figure 10:**
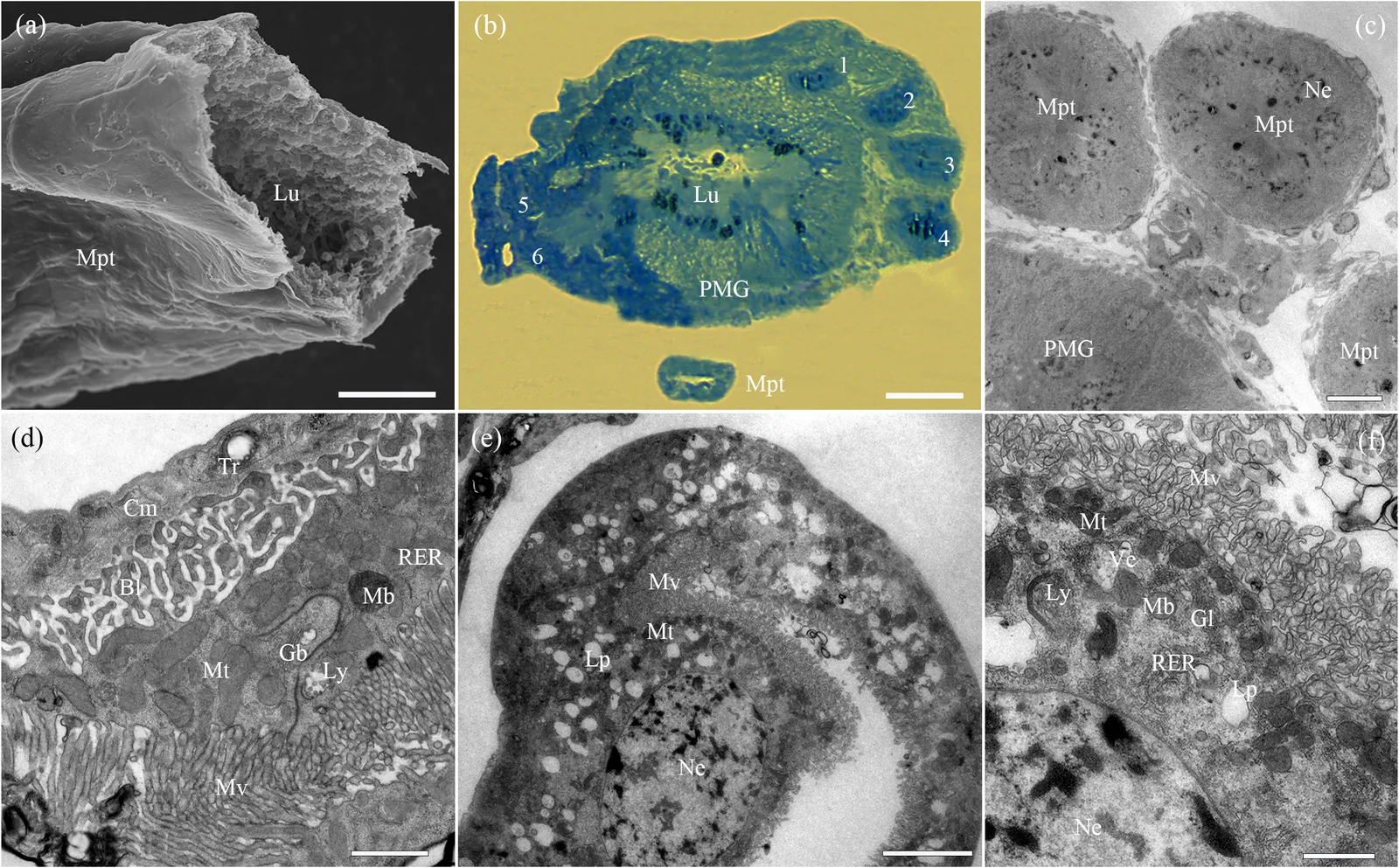
Morphology and internal structure of Malpighian tubules in *Tomicus yunnanensis*. **(a)** Fractured surface of a Malpighian tubule, showing a thin cuticle and a wide lumen; **(b)** Transverse section of the Malpighian tubule insertion site, showing four and two tubules connecting to one side (numbered); **(c)** Junction between a Malpighian tubule and the posterior midgut; **(d)** Proximal region of a Malpighian tubule; **(e)** Distal region of a Malpighian tubule; **(f)** Magnified view of the distal Malpighian tubule, showing apical cells with numerous microvilli. Bl, basal lamina; Cm, circular muscle; Gb, Golgi body; Lp, lipid droplet; Lu, lumen; Mb, multilamellar body; Mpt, Malpighian tubule; Mt, mitochondria; Mv, microvilli; Ne, nucleus; PMG, posterior midgut; RER, rough endoplasmic reticulum; Tr, tracheoles; Ve, vesicles. Scale bar, **(a, b, e)** = 5 µm; **(c)** = 10 µm; **(d, f)** = 1 µm.

Each Malpighian tubule is a long, wavy, unbranched tube with a smooth surface, a large lumen, and a uniform diameter (42.77 ± 8.15 µm), and is composed of columnar cells whose centrally located nuclei contain 8–10 nucleoli (**Figures 10a-c**). The six Malpighian tubules are arranged in two groups: four long tubules extend forward, adhering to the posterior foregut and anterior midgut before looping back toward the hindgut; the remaining two tubules are short and directed posteriorly (**Figures 3a, c, d**; **5a**; **10a**). All six tubules measure 3.84 ± 0.11 mm in length and attach to the anterior portion of the rectum, collectively forming the cryptonephridial system (**Figures 9b, f**). Internally, each Malpighian tubule is clearly divided into a proximal region and a distal region (**Figures 10d, e**). Both regions consist of a single layer of columnar cells, with a mean thickness of 4.3 ± 0.9 µm. Their cytoplasm contains abundant mitochondria, free ribosomes, and RER, as well as other organelles such as Golgi bodies, lysosomes, and electron-dense multivesicular bodies (**Figures 10d, f**). Tracheae, tracheoles, and muscle fibers are attached to the basal lamina. However, several distinct differences exist between the two regions: (1) The discrete muscle layer is thicker in the proximal region than that in the distal region; (2) The nucleus is centrally located in the proximal region, whereas in the distal region it is positioned closer to the microvilli (**Figure 10e**); (3) Mitochondria are more elongated in the proximal region than in the distal region; (4) Apical microvilli are much longer in the proximal region (1.3 µm ± 0.07 µm vs 0.7 ± 0.24 µm), but are relatively sparse and irregular in the distal region; (5) Well-organised basal infoldings are wide in the proximal region, but absent in the distal region; (6) Lipid droplets are scarce in the proximal region, but abundant in the distal region.

## 4 Discussion

This study presents a detailed morphological and ultrastructural characterisation of the alimentary canal of *T. yunnanensis*, a destructive native forest pest of southwestern China, offering anatomical insights of value to entomologists, insect physiologists, and forest pest management researchers. The alimentary canal consists of three clearly distinguishable regions: foregut, midgut, and hindgut, which closely resemble those previously reported for other scolytid beetles (Baker and Estrin, 1974; Hallberg, 1993; Díaz et al., 2000, 2003; Rubio et al., 2008; Bu and Chen, 2009; Alba-Alejandre et al., 2019), but differ from those of other polyphagous beetles, which exhibit diverse morphological adaptations to various feeding niches (Crowson, 1981; Calder, 1989; Garcia-Salazar et al., 2000; Chen et al., 2023; Özyurt Koçakoğlu et al., 2026). All scolytid beetles share common features: a proventriculus armed with chitinous plates, gastric caeca in the posterior midgut, and a cryptonephridial system associated with the rectum. A notable difference, however, is the number of Malpighian tubules: bark beetles, including *T. yunnanensis*, possess six tubules, whereas seed‑feeding beetles have only four (Alba-Alejandre et al., 2019). Based on epithelial layer thickness, the foregut is thickest, the midgut thinnest, and the hindgut of intermediate thickness, which mirrors the functional compartmentalisation of the insect alimentary canal (Silva-Olivares et al., 2003; Chapman, 2013). In *T. yunnanensis*, gut length measurements obtained by micro-CT were considerably smaller than those from dissection (**Figure 1**; **3a**; **Table 1**), likely because the non-destructive technique underestimates convoluted tube lengths. Nevertheless, micro-CT offers essential advantages for visualizing subdivisions and cross-validating with dissection. Total gut length is markedly greater in bark beetles than in other trophic guilds of scolytid beetles. For example, *T. yunnanensis* measures 15.08 mm, comparable to other bark beetles: *D. armandi i* (16.14 mm), *D. micans* (26.33 mm), *D. ponderosae* (13.57 mm), *D. pseudotsugae* (14.03 mm), *D. rufipennis* (14.96 mm), *D. terebrans* (21.93 mm) (Díaz et al., 2003; Bu and Chen, 2009). In contrast, the coffee berry borer *H. hampei* has a much shorter alimentary canal, measuring only 4.98 mm (Rubio et al., 2008). The relative proportional of foregut, midgut, and hindgut in bark beetles are approximately 15-25%, 42-52%%, and 30-40% (Díaz et al., 1998, 2000; Bu and Chen, 2009), respectively, whereas in the only seed‑feeding beetles studied, they are 16.1%, 44.2%, and 39.8% (Rubio et al., 2008). These similarities support the hypothesis that exploiting the same resource imposes ontogenetic constraints and drives morpho-functional convergence of the alimentary canal within scolytid beetles (Díaz et al., 2000; Rubio et al., 2008).

### 4.1 Foregut morphology and function in the Scolytinae

The foregut, of ectodermal origin, begins at the mouth and continues through the pharynx and esophagus. Its primary role is to transport food to the crop, which serves as a temporary storage organ (Chapman, 2013). The crop intima is lined with numerous spines that, through connections with the nervous system, are hypothesised to facilitate contraction and expansion during food storage. The muscular layers of the crop allow full distension without tearing, and the epithelial cells interposed between the muscle bundles and the cuticle prevent obstruction of fold stretching during expansion (De Sousa and Conte, 2013; Alba-Alejandre et al., 2019). In *T. yunnanensis*, the crop epithelial cells contain abundant free ribosomes, mitochondria, RER, and nucleoli, indicating a potential for local synthesis of digestive enzyme (Balogun, 1969). However, the presence of digestive enzymes in this region is generally attributed to regurgitation from the midgut or salivary glands (Baker et al., 1984; Tartar et al., 2009). It is therefore reasonable to infer that the crop of scolytids functions as a precursor site for digestion, where initial degradation of food occurs.

Immediately following the crop, the proventriculus lacks a clear external boundary. The morphology and internal structure of proventricular plates clearly reflects the texture of ingested food, including phloeophagy (bark feeding), xylomycetophagy (fungus-feeding), xylophagy (wood feeding), and spermatophagy (seed feeding) (Nobuchi, 1969; Baker and Estrin, 1974; Kirkendall et al., 2015). For example, in xylomycetophagous scolytids such as Gnathotrichus, Trypodendron, and Xyleborus, the anterior plate is short, separated from the crop, and covered only by a membranous layer, whereas the masticatory plate is elongated and bears blunt spines (Nobuchi, 1969). Even among species with similar diets, such as *D. armandi* (Bu and Chen, 2009) and *D. micans* (Díaz et al., 2000), differences in plate structure exist. In *T. yunnanensis*, each chitinous plate is typically divided into an anterior plate and a masticatory plate, conforming to Type 7 as defined by Calder (1989). The masticatory plate bears paired longitudinal brushes and rows of stopping bristles or spines that project posteriorly into the lumen, allowing food particles to pass through (Eaton, 1942). This structural feature may be characteristic of the subfamily Scolytinae (Eaton, 1942; Balogun, 1969; Crowson, 1981; Hallberg, 1993; Díaz et al., 1998, 2003; Rubio et al., 2008; Bu and Chen, 2009; Alba-Alejandre et al., 2019), and also occurs in nearly all Adephaga, most Curculionoidea, and some Polyphaga (Judd, 1952; Kissinger, 1963; Balogun, 1969; Crowson, 1981; Baker et al., 1984; de Sousa and Conte, 2013; Chen et al., 2023). During contraction of the associated circular and longitudinal muscles, these cuticular teeth compress, grind, and mix the food (Eaton, 1942). As a result, the particle size of food entering the midgut is reduced, facilitating nutrient uptake and absorption. In contrast, wood-feeding cerambycids such as *Macrotoma palmata* and *Xystocera globosa* lack a proventriculus and retain very large food particles (Mansour and Mansour-Bek, 1934), underscoring the proventriculus’s role in food processing. As noted by Díaz et al. (2003), these functional differences are reflected in the structural differentiation of the cuticle, epithelium, folds, and muscular tissue. The circular muscle layer gradually thickens from the pharynx to the proventriculus, and numerous tracheae and tracheoles overlying the muscles, together with abundant mitochondria and RER within muscle cells, supply oxygen and energy for mechanical processing (Silva-Olivares et al., 2003). In *T. yunnanensis*, several rows of spines occur along the midline between adjacent proventricular plates, blocking food particles from entering the midgut and mixing them with digestive enzymes present in the foregut, thereby prolonging food retention. This extended retention allows more time for detoxifying enzymes to degrade toxic compounds, ultimately enhancing survival capacity (Lindgren and Raffa, 2013). The relatively longer foregut and its subdivisions may help explain this functional adaptation (Díaz et al., 2000; Rubio et al., 2008; Bu and Chen, 2009). Thus, in *T. yunnanensis*, the proventriculus is responsible for limiting the passage of large and indigestible particles to the midgut.

The stomodeal valve, a transitional structure that controls the unidirectional flow of food from foregut to midgut, prevens regurgitation of intestinal contents (Chapman, 2013). In *T. yunnanensis*, the valve intima forms cup-like invaginations of the apical cytoplasmic membrane that bear short, sparse microvilli somewhat resembling those of the midgut (**Figure 4d**). This differs from the remainder of the foregut intima, which consists of a highly electron-dense epicuticle that is completely impermeable to digestive products, enzymes, and small molecules, consistent with the foregut’s lack of absorptive function (Balogun, 1969; Chapman, 2013). This suggests that the stomodeal valve and its adjacent region may possess a simple digestive function, permitting the penetration of small molecules. However, structural alterations may interfere with this function, reducing digestive efficiency and limiting the valve’s role primarily to a transitional structure that regulates food passage from the foregut to the midgut (Volf et al., 2004; Bu and Chen, 2009).

### 4.2 Midgut morphology and function in the Scolytinae

As shown in **Figure 3a** for *T. yunnanensis*, the midgut of scolytid beetles is clearly divided into anterior and posterior regions, a feature common to other Curculionidae (Crowson, 1981; Calder, 1989; Rubio et al., 2008; Candan et al., 2020). The anterior midgut is though to slow food passage, thereby facilitating mixing with digestive enzymes, while the posterior midgut, which bears abundant gastric caeca, enhances nutrient absorption efficiency (Bu and Chen, 2009). This claim, however, is based largely on structural morphology, with limited biochemical evidence. Nevertheless, the anterior and posterior regions of *T. yunnanensis* differ markedly in external shape and ultrastructure, likely reflecting functional compartmentalisation in synthesis, secretion, and absorption (Silva-Olivares et al., 2003; Caccia et al., 2019). Differences in microvilli length (**Figures 6a, c**; 7a, e) indicate regional variation in absorptive capacity, a pattern seen in *Dendroctonus* spp (Silva-Olivares et al., 2003), *Ips pini* (Nardi et al., 2002), and other insects, including *Sitophilus zeamais* (De Sousa and Conte, 2013), *S. granarius* (Baker et al., 1984), *Stomoxys calcitrans*, *Aedes gambiae* (Hecker, 1977), *Hyalophora cecropia* (Anderson and Harvey, 1966), and *Platypleura kaempferi* (Zhong et al., 2015). The presence of RER and Golgi complexes in both midgut regions of *T. yunannensis* shows active protein synthesis, consistent with the endodermal origin of the midgut (Chapman, 2013) and similar to findings in hematophagous species (Billingsley and Lehane, 1996) and *Dendroctonus* spp (Silva-Olivares et al., 2003; Bu and Chen, 2009). However, organelle distribution differs along the anteroposterior axis: secondary lysosomes and glycogen deposits are more abundant in the posterior midgut, whereas cytoplasmic vesicles are fewer there than in the anterior region. Mitochondria are also more prevalent in the apical and basal regions of posterior epithelial cells, suggesting a higher energy demand for absorption (Hecker, 1977; Silva-Olivares et al., 2003; Caccia et al., 2019). Importantly, the basal cell membrane of the posterior midgut in *T. yunnanensis* exhibits extensive invaginations forming a well-developed basal labyrinth (**Figure 7c**), which increases intercellular spaces (Billingsley and Lehane, 1996; Silva-Olivares et al., 2003; De Sousa and Conte, 2013). The thickness and length of these folds correlate with nutritional status, as greater separation indicates active digestion. This structural arrangement supports the role of the posterior midgut as the primary site for diffusion and absorption of molecules, ions, and water between hemolymph and cytoplasm. Similar observations have been reported in *Dendroctonus* spp (Silva-Olivares et al., 2003; Bu and Chen, 2009), *Ips* spp (Nardi et al., 2002), *P. kaempferi* (Zhong et al., 2015), *H. cecropia* (Anderson and Harvey, 1966), and mammalian intestinal cells (Hecker, 1977). The close association of mitochondria with basal invaginations further confirms intense absorptive activity in the posterior midgut. Together with biochemical evidence from related scolytids (e.g., high amylase levels anteriorly; abundant acid phosphatases posteriorly) (Silva-Olivares et al., 2003; De Sousa and Conte, 2013), our morphological and ultrastrucutral data indicate that the *T. yunnanensis* midgut exhibits spatial differentiation along its length, reflecting regional specialisation in synthesis, secretion, and absorption (Billingsley and Lehane, 1996; De Sousa and Conte, 2013).

Beyond their digestive and secretory functions, the midgut epithelial cells of scolytid beetles are also capable of synthesising aggregation pheromones, a role closely linked to abundant and highly organised SER (Nardi et al., 2002). However, some pheromone components, such as verbenone, can be produced by symbiotic intestinal microbes (Tittiger and Blomquist, 2016; Koski et al., 2024). In the midgut, the peritrophic membrane acts as a physical barrier excluding most bacteria and fungal spores while allowing passage of small nutrients and metabolites, indicating that the midgut is unlikely to synthesise the pheromones via microbial intermediaries (Hegedus et al., 2009). By contrast, the hindgut, harbouring diverse bacterial communities, is the more probable site for microbe-mediated production, as supported by numerous studies that have directly isolated pheromones from faeces (Brand et al., 1975; Tittiger and Blomquist, 2016; Wu et al., 2019; Koski et al., 2024). In this study, ultrastructural examination of the midgut epithelium of *T. yunnanensis* revealed a few tubular SER and some spirally arranged SER, a feature simialr to observations in *I. pini* and *D. jeffreyi*, despite minor interspecific differences in SER morphology (Nardi et al., 2002). Given the life cycle of *T. yunnanensis* and the timing of our collections (Newly mature, October), we hypothesise that the observed SER may be involved in pheromone production upon reproductive maturity. This is also inferred by Wu et al. (2019), who reported that aggregation pheromone components verbenone and trans-verbenol progressively increase during the transition from maturation feeding to the stem-infesting reproductive phase. Therefore, the presence of SER and glycogen deposits in the midgut epithelium of *T. yunnanensis* is probably indicative of de novo pheromone biosynthesis. However, definitive confirmation will require further studies, particularly in situ hybridisation to localise key biosynthetic genes (Hall et al., 2002; Koski et al., 2024).

The gastric caeca of scolytid beetles are generally located in the posterior region of the midgut and exhibit considerable interspecific variation (Schneider and Rudinsky, 1969; Baker and Estrin, 1974; Hallberg, 1993; Díaz et al., 2003; Silva-Olivares et al., 2003; Rubio et al., 2008; Bu and Chen, 2009). For example, the fungus-feeding ambrosia beetle *Xylosandrus germanus* has one tubular and one sac-shaped pair in adults, whereas larvae possess one tubular and three to five sac-shaped pairs (Yang et al., 2009). In contrast, phloem-feeding *Dendroctonus* bark beetles have far more caeca, with *D. armandi* having approximately 160 arranged in six longitudinal rows (Bu and Chen, 2009), while the coffee berry borer *H. hampei* has only two (Rubio et al., 2008; Alba-Alejandre et al., 2019). Structurally, scolytid caeca share a conserved plan, including a simple columnar epithelium, an apical microvillar brush border, and a thin basal lamina (Díaz et al., 2003; Bu and Chen, 2009; Candan et al., 2020; Özyurt Koçakoğlu et al., 2026). However, functional differentiation along the midgut is less documented in scolytids than in some other beetles (Billingsley and Lehane, 1996; Beutel et al., 2014). As shown in *S. granarius*, anterior caecal cells have numerous dark-staining apical granules, while posterior cells exhibit elongated mitochondria within their microvilli, indicating regional specialisation (Baker et al., 1984). In *T. yunnanensis*, gastric caeca arise from the posterior midgut; their epithelial cells bear microvilli and contain abundant RER, mitochondria, and Golgi complexes, but lack regenerative cells, suggesting a primary role in increasing absorptive surface and facilitating digestion. The marked differences in caecal number, morphology, arrangement, and structure between scolytids and other Coleoptera likely reflect adaptations to different diets (e.g., phloem, fungus, wood, seeds) and feeding strategies (Baker and Estrin, 1974; Crowson, 1981; Calder, 1989; Billingsley and Lehane, 1996; Beutel et al., 2014).

### 4.3 Hindgut morphology and function in the Scolytinae

The hindgut of scolytid beetles, like that of other Coleoptera, is ectodermal in origin and is typically divided into a tripartite subdivisions: the ileum, colon, and rectum. It also incorporates the pyloric valve and Malpighian tubules, and serves essential roles in faecal transport and osmoregulation (Beyenbach et al., 2010; Chapman, 2013). Structurally, the scolytid hindgut is composed of an inner cuticular intima, a middle epithelial layer, and an outer muscle layer (Hallberg, 1993; Silva-Olivares et al., 2003; Bu and Chen, 2009). However, scolytid hindguts exhibit unique structural features that distinguish them from other Coleoptera (Crowson, 1981). Among the most notable variable traits are fine spines on the cuticle surrounding the hindgut valve, which may be either present or absent irrespective of phylogenetic relatedness (Beutel et al., 2014). For example, several *Dendroctonus* species (*D. frontalis*, *D. micans*, *D. ponderosae*, *D. pseudotsugae pseudotsugae*, *D. rufipennis*, and *D. terebrans*) possess these spines (Díaz et al., 2000, 2003), whereas *D. armandi* (Bu and Chen, 2009) and *T. yunnanensis* (this study; same tribe Hylurgini) lack them, as does the more distantly related *I. typographus* (Hallberg, 1993). This variability may reflect evolutionary divergence in hindgut specialisation, potentially linked to differing feeding strategies and ecological niches. In *T. yunnanensis*, microvillus-like folds of the hindgut epithelial cells become more pronounced from the ileum towards the rectum, a pattern similar to that observed in *Dendroctonus* species (Silva-Olivares et al., 2003). This suggests that diverse macromolecules may be synthesised in this region by RER (**Figure 9c2**) and subsequently diffuse from the cytoplasm to the apical cell surface. Structurally, the exocuticle of *T. yunnanensis* is an electron-dense layer that functions as a semipermeable barrier, indicating that the hindgut is primarily involved in waste elimination rather than synthesis. A further significant observation is the presence of one or two layers of microfibrils within the endocuticle, which become more prominent from the ileum to the rectum (**Figure 9e2, f2**), suggesting increases the surface area for water and mineral ion reabsorption (Silva-Olivares et al., 2003; Bu and Chen, 2009; Chen et al., 2023). Moreover, structural changes at the pyloric valve, including altered epithelial cell shape and progressively shortened microvilli, reinforce the interpretation that the hindgut lacks a synthetic role. The epithelial cells contain few organelles and lysosomes, further supporting a primary function in waste transport rather than active synthesis. In contrast, *I. typographus* shows evidence of secretory activity in its hindgut epithelial cells, indicating further variation within the Scolytinae (Hallberg, 1993).

Furthermore, many scolytid beetles also possess a cryptonephridial system, a feature shared with several other coleopteran groups. In this arrangement, the distal ends of the Malpighian tubules are closely associated with the hindgut and enclosed by a connective tissue sheath, the perinephridial membrane (Baker and Estrin, 1974; Díaz et al., 2003; Rubio et al., 2008; Bu and Chen, 2009; Beyenbach et al., 2010; Candan et al., 2021). Ultrastructurally, the tubules consist of a single-layered epithelium of large columnar cells, as observed in *T. yunnanensis* (**Figure 10d**), characterised by an apical brush border of microvilli and a well-developed basal labyrinth, features consistent with a classical role in water and solute recovery (Beyenbach et al., 2010). However, notable differences exist between scolytids and other Coleoptera. The number of Malpighian tubules is not fixed: all known bark beetles possess six tubules in a cryptonephric arrangement (Balogun, 1969; Calder, 1989; Hallberg, 1993; Díaz et al., 2000, 2003; Bu and Chen, 2009), whereas seed-feeding beetles typically have four (Rubio et al., 2008; Alba-Alejandre et al., 2019). Other coleopteran groups exhibit either four or six tubules; for example, most Curculionidae and Cleridae have six (Baker et al., 1984; Candan et al., 2020; Chen et al., 2023), while certain chrysomelids (e.g., *Cassida palaestina*) and scarabaeid larvae have four (Garcia-Salazar et al., 2000; Lopez-Guerrero, 2002; Chen et al., 2023). Furthermore, the specific spatial association between the tubules and their insertion site can vary: in Tenebrionidae, Scolytinae, and Coccinellidae, distal tubules attach to the rectum (Crowson, 1981; Díaz et al., 2003; Bu and Chen, 2009); in others such as Chrysomelidae, Curculionoidea, Carabidae, Buprestidae, Cleridae, Cerambycidae, they attach to the colon (Calder, 1989; Garcia-Salazar et al., 2000); and in some certain species (e.g., *Epiphaneus malachiticus*), they attach to both (Candan et al., 2019). Importantly, the Malpighian tubules of *T. yunnanensis* display clear regional differentiation along their length, with distinct ultrastructural differences between the proximal and distal regions (**Figures 10d, f**). This regional differentiation is indicative of functional specialisation (Candan et al., 2021; Özyurt Koçakoğlu et al., 2026), and underpins a broad spectrum of physiological roles, including primary urine formation, solute reabsorption, aggregation pheromone biosynthesis via the hindgut–Malpighian tubule complex, detoxification, autonomous innate immunity, and water conservation (Boriden et al., 1969; Beyenbach et al., 2010; Tittiger and Blomquist, 2016). Of course, some gut-associated microbiota also participate in these functions. Therefore, additional research, especially employing functional genomics tools and function analysis of core gut microbes, is essential to verify the proposed functions within scolytid beetles (Beyenbach et al., 2010; Koski et al., 2024).

## 5 Conclusion

This study is of considerable importance, as it provides a comprehensive description of the topography, histology and ultrastructure of the alimentary canal of *T. yunnanensis*, using a synergistic combination of micro-CT reconstruction and microscopic dissection. These findings serve as a fundamental resource for understanding the adaptive gut physiology underlying food processing, detoxification, and semiochemical production in this scolytid species. In particular, structural details such as the well-developed crop, the transverse tooth lines on the proventricular intima with a robust muscle layer, and regional differences in microvilli and basal folds of midgut epithelial cells, will enhance our understanding of nutrient absorption and metabolic capacity. Furthermore, a cryptonephridial system composed of six Malpighian tubules, arranged in two groups, inserted at the midgut-hindgut junction, and attached distally to the anterior rectum, offers a valuable reference for future studies on Malpighian function. These results may also serve as a comparative framework for investigating whether midgut cells are likely to involved in de novo pheromone biosynthesis, as reported for *D. jeffreyi* (Nardi et al., 2002), and whether a conserved pheromone production strategy exists within the tribe Hylurgini (Zhao et al., 2019). In summary, by compiling fundamental biological data on *T. yunnanensis*, this study will provide a basis for understanding the alimentary canal in this pest, and for future molecular, genetic, and ecological research.

## Data Availability

The original contributions presented in this study are included in the article/**Supplementary Materials**. Further inquiries can be directed to the corresponding authors.

## References

[B1] Alba-Alejandre I. Alba-Tercedor J. Vega F. E. (2019). Anatomical study of the coffee berry borer (*Hypothenemus hampei*) using micro-computed tomography. Sci. Rep. 9, 17150. doi: 10.1038/s41598-019-53537-z 31748574 PMC6868283

[B2] Anderson E. Harvey W. R. (1966). Active transport by the cecropia midgut: II. Fine structure of the midgut epithelium. J. Cell Biol. 31, 107–134. doi: 10.1083/jcb.31.1.107 6008373 PMC2107039

[B3] Baker J. E. Woo S. M. Byrd R. V. (1984). Ultrastructural features of the gut of *Sitophilus granarius* (L.) (Coleoptera: Curculionidae) with notes on distribution of proteinases and amylases in crop and midgut. Can. J. Zool. 62, 1251–1259. doi: 10.1139/z84-18134819996

[B4] Baker W. V. Estrin C. L. (1974). The alimentary canal of *Scolytus multistriatus* (Coleoptera: Scolytidae): a histological study. Can. Entomol. 106, 673–686. doi: 10.4039/Ent106673-7

[B5] Balogun R. A. (1969). Digestive enzymes of the alimentary canal of the larch bark beetle *Ips cembrae* Heer. Comp. Biochem. Physiol. 29, 1267–1270. doi: 10.1016/0010-406X(69)91033-0

[B6] Beutel R. G. Friedrich F. Yang X.-K. Ge S.-Q. (2014). Insect Morphology and Phylogeny: A Textbook for Students of Entomology (Berlin: Walter de Gruyter).

[B7] Beyenbach K. W. Skaer H. Dow J. A. T. (2010). The developmental, molecular, and transport biology of Malpighian tubules. Annu. Rev. Entomol. 55, 351–374. doi: 10.1146/annurev-ento-112408-085512 19961332

[B8] Billingsley P. F. Lehane M. J. (1996). “ Structure and ultrastructure of the insect midgut,” in Biology of the Insect Midgut. Eds. Lehane M. J. Billingsley P. F. ( Springer Netherlands, Dordrecht), 3–30.

[B9] Boriden J. H. Nair K. K. Slater C. E. (1969). Synthetic juvenile hormone: induction of sex pheromone production in *Ips confusus*. Science 166, 1626–1627. doi: 10.1126/science.166.3913.1626 17758717

[B10] Brand J. M. Bracke J. W. Markovetz A. J. Wood D. L. Browne L. E. (1975). Production of verbenol pheromone by a bacterium isolated from bark beetles. Nature 254, 136–137. doi: 10.1038/254136a0 804144

[B11] Bu S.-H. Chen H. (2009). The alimentary canal of *Dendroctonus armandi* Tsai and Li (Coleoptera: Curculionidae: Scolytinae). Coleopt. Bull. 63, 485–496. doi: 10.1649/1190.142369796

[B12] Caccia S. Casartelli M. Tettamanti G. (2019). The amazing complexity of insect midgut cells: types, peculiarities, and functions. Cell Tissue Res. 377, 505–525. doi: 10.1007/s00441-019-03076-w 31359140

[B13] Calder A. A. (1989). The alimentary canal and nervous system of Curculionoidea (Coleoptera): Gross morphology and systematic significance. J. Nat. Hist. 23, 1205–1265. doi: 10.1080/0022293890077067137339054

[B14] Candan S. Özyurt Koçakoğlu N. Erbey M. (2019). Morphology and histology of the alimentary canal of *Epiphaneus malachiticus* Boheman 1842 (Coleoptera, Curculionidae). Entomol. Rev. 99, 326–336. doi: 10.1134/S0013873819030059

[B15] Candan S. Özyurt Koçakoğlu N. Güllü M. Çağlar Ü. (2020). Anatomical and histological studies of the alimentary canal of adult maize leaf weevil, *Tanymecus dilaticollis* Gyllenhal 1834(Coleoptera: curculionidae). Microsc. Res. Tech. 83, 1153–1162. doi: 10.1002/jemt.23507 32483898

[B16] Candan S. Özyurt Koçakoğlu N. Serttaş A. (2021). Histoanatomy of Malpighian tubules and the digestive tract of adult of biocontrol agent *Calosoma sycophanta* L. (Coleoptera: Carabidae). Int. J. Trop. Insect Sci. 41, 1373–1386. doi: 10.1007/s42690-020-00331-430311153

[B17] Chapman R. F. (2013). The Insects: Structure and Function (5th Edition) (Cambridge: Cambridge University Press).

[B18] Chen L. Liu M. Di Giulio A. Chen X. Sabatelli S. Wang W. . (2023). Morphological study of the alimentary canal and Malpighian tubules in the adult of the pollen beetle *Meligethes (Odonthogethes) chinensis* (Coleoptera: Nitidulidae: Meligethinae). Insects 14, 298. doi: 10.3390/insects14030298 36975983 PMC10057167

[B19] Crowson R. A. (1981). The Biology of the Coleoptera (London, UK: Academic press).

[B20] De Sousa G. Conte H. (2013). Midgut morphophysiology in *Sitophilus zeamais* Motschulsky 1855(Coleoptera: curculionidae). Micron 51, 1–8. doi: 10.1016/j.micron.2013.06.001 23810449

[B21] Díaz E. Arciniega O. Sánchez L. Cisneros R. Zúñiga G. (2003). Anatomical and histological comparison of the alimentary canal of *Dendroctonus micans*, *D. ponderosae*, *D. pseudotsugae pseudotsugae*, *D. rufipennis*, and *D. terebrans* (Coleoptera: Scolytidae). Ann. Entomol. Soc Am. 96, 144–152. doi: 10.1603/0013-8746(2003)096[0144:aahcot]2.0.co;2

[B22] Díaz E. Cisneros R. Zúñiga G. (2000). Comparative anatomical and histological study of the alimentary canal of the *Dendroctonus frontalis* (Coleoptera: Scolytidae) complex. Ann. Entomol. Soc Am. 93, 303–311. doi: 10.1603/0013-8746(2000)093[0303:caahso]2.0.co;2

[B23] Díaz E. Cisneros R. Zuñiga G. Uria-Galicia E. (1998). Comparative anatomical and histological study of the alimentary canal of *Dendroctonus parallelocollis*, *D. rhizophagus*, and *D. valens* (Coleoptera: Scolytidae). Ann. Entomol. Soc Am. 91, 479–487.

[B24] Eaton C. B. (1942). The anatomy and histology of the proventriculus of *Ips radiatae* Hopkins (Coleoptera: Scolytidae). Ann. Entomol. Soc Am. 35, 41–49. doi: 10.1093/aesa/35.1.41

[B25] Garcia-Salazar C. Gildow F. E. Fleischer S. J. Cox-Foster D. Lukezic F. L. (2000). Alimentary canal of adult *Acalymma vittata* (Coleoptera: Chrysomelidae): Morphology and potential role in survival of Erwinia tracheiphila (enterobacteriaceae). Can. Entomol. 132, 1–13. doi: 10.4039/Ent1321-1

[B26] Haider K. Abbas D. Galian J. Ghafar M. A. Kabir K. Ijaz M. . (2025). The multifaceted roles of gut microbiota in insect physiology, metabolism, and environmental adaptation: implications for pest management strategies. World J. Microbiol. Biotechnol. 41, 75. doi: 10.1007/s11274-025-04288-9 40011281

[B27] Hall G. M. Tittiger C. Blomquist G. J. Andrews G. L. Mastick G. S. Barkawi L. S. . (2002). Male Jeffrey pine beetle, *Dendroctonus jeffreyi*, synthesizes the pheromone component frontalin in anterior midgut tissue. Insect Biochem. Mol. Biol. 32, 1525–1532. doi: 10.1016/S0965-1748(02)00073-5 12530220

[B28] Hallberg E. (1993). Fine structure of the hindgut of *Ips typographus* (Coleoptera: Scolytidae). Entomol. Gen. 18, 25–36. doi: 10.1127/entom.gen/18/1993/2542579809

[B29] Hecker H. (1977). Structure and function of midgut epithelial cells in Culicidae mosquitoes (Insecta, Diptera). Cell Tissue Res. 184, 321–341. doi: 10.1007/BF00219894 21747

[B30] Hegedus D. Erlandson M. Gillott C. Toprak U. (2009). New insights into peritrophic matrix synthesis, architecture, and function. Annu. Rev. Entomol. 54, 285–302. doi: 10.1146/annurev.ento.54.110807.090559 19067633

[B31] Judd W. W. (1952). The proventriculus of the diamond beetle, *Entimus nobilis* Oliv. (Coleoptera: Curculionidae). Can. Entomol. 84, 181–184. doi: 10.4039/Ent84181-6

[B32] Kirkendall L. R. Biedermann P. H. W. Jordal B. H. (2015). “ Evolution and diversity of bark and ambrosia beetles,” in Bark Beetles. Eds. Vega F. E. Hofstetter R. W. ( Academic Press, San Diego), 85–156.

[B33] Kirkendall L. R. Faccoli M. Ye H. (2008). Description of the Yunnan shoot borer, *Tomicus yunnanensis* Kirkendall & Faccoli sp. n. (Curculionidae, Scolytinae), an unusually aggressive pine shoot beetle from southern China, with a key to the species of *Tomicus*. Zootaxa 1819, 25–39. doi: 10.11646/zootaxa.1819.1.2

[B34] Kissinger D. G. (1963). The proventricular armature of Curculionidae (Coleoptera). Ann. Entomol. Soc Am. 56, 769–771. doi: 10.1093/aesa/56.6.769

[B35] Koski T.-M. Zhang B. Wickham J. D. Bushley K. E. Blanchette R. A. Kang L. . (2024). Chemical interactions under the bark: bark-, ambrosia-, and wood-boring beetles and their microbial associates. Rev. Environ. Sci. Bio/Technol. 23, 923–948. doi: 10.1007/s11157-024-09709-z30311153

[B36] Lieutier F. Långström B. Faccoli M. (2015). “ The genus *tomicus*,” in Bark Beetles: Biology and Ecology of Native and Invasive Species. Eds. Vega F. E. Hofstetter R. W. ( Academic Press, San Diego), 371–426.

[B37] Lindgren B. S. Raffa K. F. (2013). Evolution of tree killing in bark beetles (Coleoptera: Curculionidae): trade-offs between the maddening crowds and a sticky situation. Can. Entomol. 145, 471–495. doi: 10.4039/tce.2013.27

[B38] Liu F. Wu C. Zhang S. Kong X. Zhang Z. Wang P. (2019). Initial location preference together with aggregation pheromones regulate the attack pattern of *Tomicus brevipilosus* (Coleoptera: Curculionidae) on *Pinus kesiya*. Forests 10, 156. doi: 10.3390/f1002015630654563

[B39] Liu J. Zhang M. Qian L. Wang Z. Li Z. (2025). Electrophysiology and behavior of *Tomicus yunnanensis* to *Pinus yunnanensis* volatile organic compounds across infestation stages in southwest China. Forests 16, 1178. doi: 10.3390/f1607117830654563

[B40] Liu H. Zhang Z. Ye H. Wang H. Clarke S. R. Lu J. (2010). Response of *Tomicus yunnanensis* (Coleoptera: Scolytinae) to infested and uninfested *Pinus yunnanensis* Bolts. J. Econ. Entomol. 103, 95–100. doi: 10.1603/EC09080 20214373

[B41] Lopez-Guerrero Y. (2002). Anatomy and histology of the digestive system of *Cephalodesmius armiger* Westwood (Coleoptera, Scarabaeidae, Scarabaeinae). Coleopt. Bull. 56, 97–106. doi: 10.1649/0010-065x(2002)056[0097:aahotd]2.0.co;2

[B42] Lu R. C. Wang H. B. Zhang Z. Byers J. A. Jin Y. J. Wen H. F. . (2012). Coexistence and competition between *Tomicus yunnanensis* and *T. minor* (Coleoptera: Scolytinae) in Yunnan pine. Psyche, 1–6. doi: 10.1155/2012/185312

[B43] Mansour K. Mansour-Bek J. J. (1934). The digestion of wood by insects and the supposed role of micro-organisms. Biol. Rev. 9, 363–382. doi: 10.1111/j.1469-185X.1934.tb01252.x40046247

[B44] Nardi J. B. Young A. G. Ujhelyi E. Tittiger C. Lehane M. J. Blomquist G. J. (2002). Specialization of midgut cells for synthesis of male isoprenoid pheromone components in two scolytid beetles, *Dendroctonus jeffreyi* and *Ips pini*. Tissue Cell. 34, 221–231. doi: 10.1016/S0040-8166(02)00004-6 12176306

[B45] Nobuchi A. (1969). A comparative morphological study of the proventriculus in the adult of the super-family Scolytoidea (Coleoptera). Bull. Gov. For. Exp. Sta. 224, 39–110.

[B46] Özyurt Koçakoğlu N. Çağlar Ü. Arslan H. Candan S. (2026). Histological and electron microscopy observations on the alimentary canal and Malpighian tubules of the strawberry pest *Coraebus elatus* (Fabricius 1787) (Coleoptera, buprestidae). Acta Zool. 107, 337–347. doi: 10.1111/azo.1256040046247

[B47] Rubio G. J. D. Bustillo P. A. E. Vallejo E. L. F. Acuña Z. J. R. Benavides M. P. (2008). Alimentary canal and reproductive tract of *Hypothenemus hampei* (Ferrari) (Coleoptera: Curculionidae, Scolytinae). Neotrop. Entomol. 37, 143–151. doi: 10.1590/S1519-566X2008000200006 18506292

[B48] Schneider I. Rudinsky J. A. (1969). Anatomical and histological changes in internal organs of adult *Trypodendron lineatum*, *Gnalhotrichus retusus*, and *G. sulcatus* (Coleoptera: Scolytidae). Ann. Entomol. Soc Am. 62, 995–1003. doi: 10.1093/aesa/62.5.995

[B49] Silva-Olivares A. Díaz E. Shibayama M. Tsutsumi V. Cisneros R. Zúñiga G. (2003). Ultrastructural study of the midgut and hindgut in eight species of the genus *Dendroctonus* Erichson (Coleoptera: Scolytidae). Ann. Entomol. Soc Am. 96, 883–900. doi: 10.1603/0013-8746(2003)096[0883:usotma]2.0.co;2

[B50] Tartar A. Wheeler M. M. Zhou X. Coy M. R. Boucias D. G. Scharf M. E. (2009). Parallel metatranscriptome analyses of host and symbiont gene expression in the gut of the termite *Reticulitermes flavipes*. Biotechnol. Biofuels 2, 25. doi: 10.1186/1754-6834-2-25 19832970 PMC2768689

[B51] Tittiger C. Blomquist G. J. (2016). “ Pheromone production in pine bark beetles,” in Pine Bark Beetles, Advances in Insect Physiology. Eds. Tittiger C. Blomquist G. J. ( Academic Press, London), 235–263.

[B52] Volf P. Hajmova M. Sadlova J. Votypka J. (2004). Blocked stomodeal valve of the insect vector: similar mechanism of transmission in two trypanosomatid models. Int. J. Parasitol. 34, 1221–1227. doi: 10.1016/j.ijpara.2004.07.010 15491584

[B53] Wang L. Li C. Luo Y. Wang G. Dou Z. Haq I. U. I. . (2023). Current and future control of the wood-boring pest *Anoplophora glabripennis*. Insect Sci. 30, 1534–1551. doi: 10.1111/1744-7917.13187 36944595

[B54] Wu C. X. Liu F. Zhang S. F. Kong X. B. Zhang Z. (2019). Semiochemical regulation of the intraspecific and interspecific behavior of *Tomicus yunnanensis* and *Tomicus minor* during the shoot-feeding phase. J. Chem. Ecol. 45, 227–240. doi: 10.1007/s10886-019-01048-6 30796677

[B55] Yang Q.-F. Li Q. Zhi Y.-R. (2009). Anatomical study of the alimentary canal of *Xylosandrus germanus*. Entomological Knowledge 46, 623–626.

[B56] Yavorskaya M. I. Anton E. Jałoszyński P. Polilov A. Beutel R. G. (2018). Cephalic anatomy of Sphaeriusidae and a morphology-based phylogeny of the suborder Myxophaga (Coleoptera). Syst. Entomol. 43, 777–797. doi: 10.1111/syen.1230440046247

[B57] Ye H. (1991). On the bionomy of *Tomicus piniperda* (L.) (Col. Scolytidae) in the Kunming region of China. J. Appl. Entomol. 112, 366–369. doi: 10.1111/j.1439-0418.1991.tb01069.x40046247

[B58] Ye H. (1994). Influence of temperature on the experimental population of the pine shoot beetle, *Tomicus piniperda* (L.) (Col. Scolytidae). J. Appl. Entomol. 117, 190–194. doi: 10.1111/j.1439-0418.1994.tb00724.x40046247

[B59] Yu L. Zhan Z. Zhou Q. Gao B. Ren L. Huang H. . (2022). Climate drivers of pine shoot beetle outbreak dynamics in Southwest China. Remote Sens. 14, 2728. doi: 10.3390/rs1412272830654563

[B60] Zhao T. Ganji S. Schiebe C. Bohman B. Weinstein P. Krokene P. . (2019). Convergent evolution of semiochemicals across Kingdoms: bark beetles and their fungal symbionts. ISME J. 13, 1535–1545. doi: 10.1038/s41396-019-0370-7 30770902 PMC6776033

[B61] Zhong H. Zhang Y. Wei C. (2015). Morphology and ultrastructure of the alimentary canal of the cicada *Platypleura kaempferi* (Hemiptera: Cicadidae). Entomol. Sci. 18, 340–352. doi: 10.1111/ens.1211540046247

