# Supplementary material for: Micro-CT reconstruction and structural morphology of the alimentary canal in adult Tomicus yunnanensis (Coleoptera: Curculionidae: Scolytinae)
Source: Front Physiol. 2026 Sep 7;17:1922960. doi: 10.3389/fphys.2026.1922960 (PMC13590421; doi:10.3389/fphys.2026.1922960)
Supplement: Supplementary file 1 [file SupplementaryFile1.docx]

Supplementary Material

# 1 Supplementary Figures

**Supplementary Figure 1.** Transverse micro‑CT sections of the alimentary canal in adult Tomicus yunnanensis: female (top, non‑starved) and male (bottom, starved). (a, d) Terminal proventriculus; (b, e) middle proventriculus; (c, f) distal proventriculus.

**Supplementary Figure 2.** Ultrastructure of the peritrophic membrane, revealing a fibrous matrix enveloping the food bolus.

**Supplementary Figure 3.** Interwoven spiral smooth and rough endoplasmic reticulum in the anterior midgut of *Tomicus yunnanensis*.
